# Unveiling Weevil Diversity Drivers and Cryptic Species on the Qinghai–Xizang Plateau

**DOI:** 10.3390/insects17010120

**Published:** 2026-01-21

**Authors:** Jinliang Ren, Jiahua Xing, Xuan Liu, Runzhi Zhang

**Affiliations:** 1 State Key Laboratory of Animal Biodiversity Conservation and Integrated Pest Management, Institute of Zoology, Chinese Academy of Sciences, Beijing 100101, China; renjinliang@ioz.ac.cn (J.R.); xingjh@smail.nju.edu.cn (J.X.); 2 Lab of Animal Behavior and Conservation, School of Life Sciences, Nanjing University, Nanjing 210023, China; 3 College of Life Science, University of Chinese Academy of Sciences, Beijing 100049, China

**Keywords:** Curculionoidea, cryptic diversity, DNA barcode, environmental heterogeneity, species delimitation

## Abstract

Understanding patterns and mechanisms of species diversity is one fundamental issue in biogeography and ecology. As a critical region for biodiversity, the Qinghai-Xizang Plateau (QXP) still has unclear distribution patterns and drivers for cryptic, understudied taxa such as Curculionoidea. Here, we collected the distribution data of Curculionoidea on the QXP to analyze their diversity patterns and influencing factors, and compiled a DNA barcode dataset to uncover cryptic diversity. This comprehensive dataset encompasses 671 Curculionoidea species across 223 genera, demonstrating a level of diversity that surpasses that of certain vertebrate groups. We also observed an unbalanced biogeographic pattern of diversity, with a concentration of species in the eastern and southern regions and a scarcity in the northern and central areas of QXP. Further analysis showed that the elevation range is the most important factor influencing the diversity of Curculionoidea. In addition, based on 1147 COI-5′ barcode sequences from 217 species, we found that 11 morphological species may contain cryptic species based on DNA barcode datadset. Our findings significantly enhance the current understanding of cryptic biodiversity patterns among understudied taxa in the QXP, while simultaneously highlighting persistent knowledge gaps in characterizing the plateau’s full ecological complexity.

## Simple Summary

Based on a study of weevils (Curculionoidea) on the Qinghai-Xizang Plateau, this research found their species diversity to be higher than that of vertebrates, with an uneven distribution concentrated in the eastern and southern regions. Elevation range emerged as the most critical factor influencing diversity. DNA barcode analysis also suggested the potential existence of cryptic species within 11 morphological species. These findings enhance the understanding of biodiversity in understudied taxa on the plateau and highlight the need for further multidisciplinary research.

## 1. Introduction

Understanding geographic distribution patterns and their drivers has long been a core focus in biogeography and ecology [1]. It is critical both for deciphering species’ evolutionary histories and for informing effective biodiversity conservation strategies [2]. Cryptic species with different evolution history are usually classified as the same species due to morphological convergence [3,4,5]. Mechanisms leading to cryptic diversity are various, comprising recent divergence, niche conservatism and morphological convergence [6]. The discovery of cryptic species carries profound implications for biogeographic reconstructions [6], and conservation prioritization [7].

Mountains are also considered as the biodiversity conservation hotspot owing to their coverage of 85% of amphibian, bird, and mammal species with only 25% of the Earth’s surface [1]. They are also considered the “cradles” with rapid species origination, “museums” with ancient species preservation, and “tombs” with high species extinction rate [1]. The Qinghai-Xizang Plateau (QXP)—the world’s largest and highest plateau (average elevation > 4000 m) [8] —is renowned for its complex climate systems and exceptional biodiversity. Within its boundaries, the entirely contained Himalayan Mountains and the Hengduan Mountains (whose main body lies within the southeastern QXP) are both designated among the globe’s 25 biodiversity hotspots [2]. A study of 555 endemic plants in China revealed that the eastern margin of the QXP acts as both a cradle and an “evolutionary frontier” for these plants [9]. Another study, encompassing 7822 Hemiptera species across China, suggested that the Eastern Himalayas and Hengduan Mountains may serve as both cradles and museums for these species [10]. However, it needs further evidences from more taxa to elucidate the general role of the QXP in shaping the high biodiversity across taxa.

The extraordinary biodiversity of the QXP has been shaped by interconnected geological and climatic mechanisms [11,12,13]. Tectonic uplift created geographic barriers (e.g., mountains and valleys) that restricted gene flow, driving population divergence and allopatric diversification [14,15,16,17]. Moreover, orogenic activity and climatic cooling contribute to the heterogeneous habitats, including shrublands, grasslands, and deserts. And the diverse habitats facilitated adaptive radiation through ecological niche partitioning [18,19,20]. During Quaternary glaciations, ice-free refugia across the QXP and adjacent mountains preserved relict lineages and promoted in situ speciation [21,22,23,24,25], serving as reservoirs for postglacial recolonization [19]. Furthermore, Pleistocene climatic oscillations amplified genetic differentiation through repeated population fragmentation and reconnection, as documented by isolation-by-environment dynamics [12,13,15,26].

There are approximately 5.5 million insect species globally, which is one of the most diverse and widely distributed groups on Earth [27]. Curculionoidea is one of the largest superfamily of Coleoptera, comprising over 5800 genera and more than 62,000 species documented worldwide [28]. Numerous species within this group are significant agricultural and forestry pests, such as *Anthonomus grandis* Boheman [29], *Lissorhoptrus oryzophilus* Kuschel [30], and *Rhynchophorus ferrugineus* (Oliver) [31]. Over the past four decades, numerous studies have conducted extensive descriptions of weevil species from QXP, e.g., [32,33]. Despite extensive taxonomic studies, key aspects of this diverse group remain poorly investigated, such as its biodiversity patterns and cryptic species diversity, which reveals significant gaps in our ecological understanding of these organisms.

Here, we integrated distribution records and DNA barcodes of Curculionoidea from QXP to (i) provide a comprehensive summary of the species diversity of Curculionoidea; (ii) analyze species richness spatial patterns and identify diversity hotspots among Curculionoidea insects; (iii) test the relative importance of environmental variables in shaping richness patterns of Curculionoidea; (iv) explore the cryptic diversity of the weevils in the QXP region.

## 2. Materials and Methods

### 2.1. Study Area and Distribution Dataset of the Weevil Insects

The QXP, spanning 26°00′12″~39°46′50″ N and 73°18′52″~104°46′59″ E, extends 2945 km from east to west, and 1532 km from north to south, covering an area of about 2.6 million km^2^, which accounts for 26.8% of China’s total land area [8]. It is surrounded by high mountains, with the Kunlun Mountains in the north, the Himalayas Mountains in the south, the Karakoram Mountains in the west, and the steep Hengduan Mountains in the east (Figure S1). The vector data for the map of the QXP was derived from [34]. For follow-up analysis, we divided the map of the QXP into 0.5°× 0.5° grid units in ArcGIS 10.7 (ESRI, Inc., Redlands, CA, USA), subsequently.

The species list was compiled based on the classification system from [35]. The distribution records of species were mainly obtained from the following sources: (i) 4000 specimens collected from the QXP during 2018–2022 (Table S1); (ii) 4194 specimens examined in the Institute of Zoology, Chinese Academy of Sciences; (iii) published data (e.g., [32,33,36,37,38]); (iv) the Zoological Record database (http://webofknowledge.com/ZOOREC, accessed on 1 January 2023). For distribution records that provide latitude and longitude in the original data source, the geographic reference data are directly retained. For those records that provide only the name of the place, their geographic coordinates are determined using Google Maps.

### 2.2. Environmental Data

In order to estimate the relative effects of contemporary climate, historical climate change and habitat heterogeneity on species richness of Curculionoidea insects, we divided the environmental factors into five categories. (i) Energy availability, including mean annual temperature (MEAT), mean temperature of warmest quarter (MTWAQ), mean temperature of coldest quarter (MTCQ), minimum temperature of coldest month (MTCM), and maximum temperature of warmest month (MTWM). (ii) Water availability, including mean annual precipitation (MAP), precipitation of warmest quarter (PWAQ), and precipitation of coldest quarter (PCQ). (iii) Contemporary climate stability, including mean diurnal range (MDR), and precipitation seasonality (PS). (iv) Historical climate change, including Tano (anomaly of MAT between the Last Glacial Maximum and present), and Pano (anomaly of MAP between the Last Glacial Maximum and present). (v) Habitat heterogeneity, including normalized difference vegetation index (NDVI), and elevation range (ELE).

Both modern temperature and precipitation data were retrieved from the WorldClim website at a spatial resolutions of 30 arc-seconds [39]. Past climate data (Tano and Pano) were reconstructed using the MIROC-ESM model [40]. ELE was defined as the difference between the maximum elevation and the minimum elevation within each grid cell using a digital elevation model derived from the Global 30 Arc-Second Elevation Dataset (GTOPO30) at a resolution of 1 km (https://eros.usgs.gov/elevation-products, accessed on 1 June 2024). NDVI data were retrieved from the Environment and Ecology Scientific Data Center of western China, National Natural Science Foundation of China (http://westdc.westgis.ac.cn).

Environmental data of all grids with a spatial resolution of 0.5° × 0.5° were extracted by calculating the average value for each grid cell. Data extraction was performed using ArcGIS 10.7 (ESRI, Inc., Redlands, CA, USA) and R x64 4.3.1 (http://www.r-project.org/).

### 2.3. Data Analysis

Since the frequency distribution of species richness (i.e., the total number of species within each grid cell) conforms to a Poisson distribution, we employed Generalized Linear Models (GLMs) to test the relationship between the richness of Curculionoidea species and environmental variables. Therefore, the GLMs with quasipoisson errors [41] were used to assess the explanatory power of the environmental variables using the glm function in R x64 4.3.1 (http://www.r-project.org/). Consistent with previous studies [10,42,43], we used the adjusted R^2^_adj_ (%) to measure the explanatory power of each variable. Modified *t*-test was used to test the significance level of each regression coefficient, thereby eliminating the influence of spatial autocorrelation in the significance test [44].

In addition to GLMs analysis, we also employed Random Forest, another method for exploring the relationship between species richness and environmental factors, to evaluate the relative importance of each variable. Random Forest is not only insensitive to spatial autocorrelation but can also handle nonlinear relationships between species richness and variables [45,46]. Based on the Random Forest function in R x64 4.3.1, we obtained the relative importance values of each environmental factor in terms of mean decrease in node purity.

Considering most contemporary climate variables were highly correlated (Figure S2), we also conducted analyses through multivariate approaches. To eliminate dimensional effects between data features, all environmental variables were standardized. And to avoid strong multicollinearity among variables, we calculated variable correlation results (Figures S2 and S3) and excluded variables with correlations > 0.8, ultimately retaining variables with VIF (variance inflation factor) < 4. We then constructed different regression models and determined the optimal combination of environmental variables using the BIC (Bayesian Information Criterion) value. Subsequently, we decomposed the shared R^2^ (proportion of variance explained) using glmm.hp to clarify the independent contributions of different explanatory variables to the response variable in the optimal combined model [47]. Finally, we validated the results using a random forest model [45,46].

### 2.4. Taxon Sampling and Data Collection

Fieldwork was conducted in the QXP from 2018 to 2022. For each collected specimen, the longitude, latitude, and altitude were recorded. Subsequently, the specimens were preserved in 100% ethanol at −20 °C until further analysis. All collected specimens were identification by Dr. Li Ren and the authors. To enhance the representativeness of our study, we aimed to collect specimens from various locations across the QXP, ensuring maximum coverage and facilitating comprehensive observations of intraspecific variation. However, due to constraints in the field collection conditions, samples were primarily collected from the southern and eastern edges of the plateau (Figure S4). All voucher specimens were deposited in the Institute of Zoology (IZCAS), Chinese Academy of Sciences, Beijing, China.

We retrieved sequences of QXP weevils (up to August 2023) from the BOLD database. To refine our dataset, we applied filters to exclude sequences that were (i) shorter than 600 bp, (ii) containing degenerate or missing bases, (iii) having ambiguous bases, and (iv) unidentified at the species level. The removal of duplicate sequences was performed for each species. Subsequently, all sequences were translated into amino acids using MEGA 7 to verify and prevent the occurrence of stop codons.

### 2.5. DNA Extraction, PCR Amplification, Sequencing and Alignment

DNeasy Blood & Tissue Kits (QIAGEN, Hilden, Germany) were used to extract the total DNA of the sample. Polymerase chain reaction (PCR) amplifications for COI sequences were performed using the primers LCO1490 (GGTCAACAAATCATAAAGATATTGG) and HCO2198 (TAAACTTCAGGGTGACCAAAAAATCA). PCR reaction mixes (25 mL) contained 12.5 μL 2× Taq PCR MasterMix (Tiangen Biotech Co., Ltd., Beijing, China), 1 μL of each forward and reverse primer (Sangon Biotech Co. Ltd., Shanghai, China), 2 μL total undiluted DNA template, and 8.5 μL dd H_2_O. The PCR profile was as follows: 94 °C for 2 min, first cycle set (five cycles): 94 °C for 40 s, 45 °C for 40 s, and 72 °C for 60 s. Second cycle set (35 cycles): 94 °C for 40 s, 51 °C for 40 s, and 72 °C for 60 s, followed by a final elongation at 75 °C for 5 min [48]. The PCR products were visualized through 1% agarose gel electrophoresis in TAE buffer. Subsequently, the successful PCR products were sent to the Beijing Genomics Institute (BGI, Shenzhen, China) for sequencing. The raw data were then assembled using SeqMan 7.1. Next, all sequences were translated into amino acids using MEGA 7 to verify and prevent the occurrence of stop codons. Finally, all sequences were aligned in MEGA 7.

### 2.6. Genetic Distance and Phylogenetic Analysis

The Kimura 2-parameter (K2P) model in MEGA 7 was used to calculate the intraspecific genetic distances (intra-GD) and interspecific genetic distances (inter-GD) [49]. Origin 2018 was used to visualize and represent the distribution of genetic distances through histograms and scatter plots [50]. Phylogenetic inference analyses were conducted using maximum likelihood (ML) method. The optimal nucleotide substitution models and ML analyses were selected based on the Akaike information criterion (AIC) via the IQ-TREE web server [51]. The ML analyses were performed with the following settings: ultrafast bootstrap, ML + rapid bootstrap [52], 1000 replicates, and the GTR + F + I + G4 model.

### 2.7. Species Delimitation and Cryptic Diversity Discovery

The four species delimitation methods, namely Automatic Barcode Gap Discovery (ABGD), Assemble Species by Automatic Partitioning (ASAP), jMOTU, and the Bayesian Poisson tree processes (bPTP), were used to evaluate the species boundaries and identify cryptic species. The ABGD analyses identify a divergence gap that corresponds to the differentiation between intraspecific and interspecific distances [53]. These analyses were performed at the ABGD tool within the SpartExplorer platform (https://spartexplorer.mnhn.fr/, accessed on 7 March 2024) with the following parameter: a relative gap width of X = 1.5, K2P distance metric, and intraspecific divergence (*p*) values ranging from 0.005 to 0.1. The ASAP analyses are a method to build species partitions from single locus sequence alignments [54], and were performed on the ASAP tool within the SpartExplorer platform (https://spartexplorer.mnhn.fr/, accessed on 7 March 2024) using the K2P distance metric. Partition with the smallest score is considered as the final result of the species delimitation. The jMOTU 1.0.7 analyses can efficiently and robustly identify molecular classification groups present in survey datasets within a short timeframe [55]. These analyses were performed using the jMOTU program with parameters set as follows: MOTU definition from 1 to 50, a low BLAST identity filter at 97%, a minimum sequence length percentage of 95%, and the number of processors used in Magablast set to 4. The bPTP analyses consider the number of substitutions between branching and speciation as independent events [56] and were performed on the web server (http://species.h-its.org/ptp/, accessed on 24 March 2024) using the following settings: 500,000 MCMC generations, with the initial 20% of trees discarded as burn-in. When same morphospecies were grouped into more than one MOTUs by three or four more species delimitation methods, we believe that there is cryptic species diversity.

## 3. Results

### 3.1. Species Diversity of Curculionoidea Insects in the QXP

**Figure 1:**
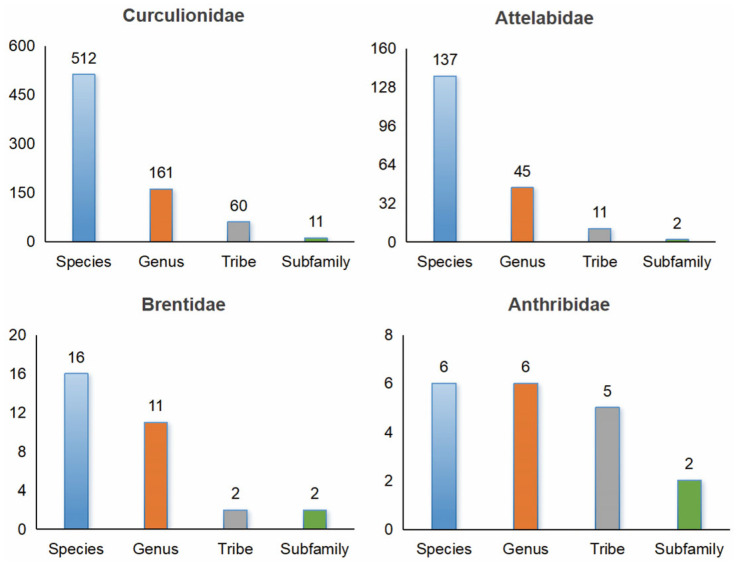
Species diversity of Curculionoidea on the QXP.

Our integrated analysis confirms the presence of 671 Curculionoidea species on the QXP, belonging to 223 genera, 78 tribes, 17 subfamilies and 4 families (Figure 1). Among all Curculionoidea groups, the Curculionidae, represented by 512 species, was the most abundant family, accounting for 76.3% of the total species. The total number of species of global birds, reptiles, amphibians, and mammals (11,185, 12,386, 8863, and 6736 species, respectively) amounts to approximately 39,170 species (https://ourworldindata.org/how-many-species-are-there, accessed on 22 June 2025), which is only about 63.1% of the known weevil species worldwide [28]. In contrast, on the QXP, which is the focus of this study, the recorded total of these four vertebrate groups is 1231 species, while the number of weevil species exceeds half of that (54.5%) and also surpasses half of the Hemipteran species (57.4%). (Figure S5).

**Figure 2:**
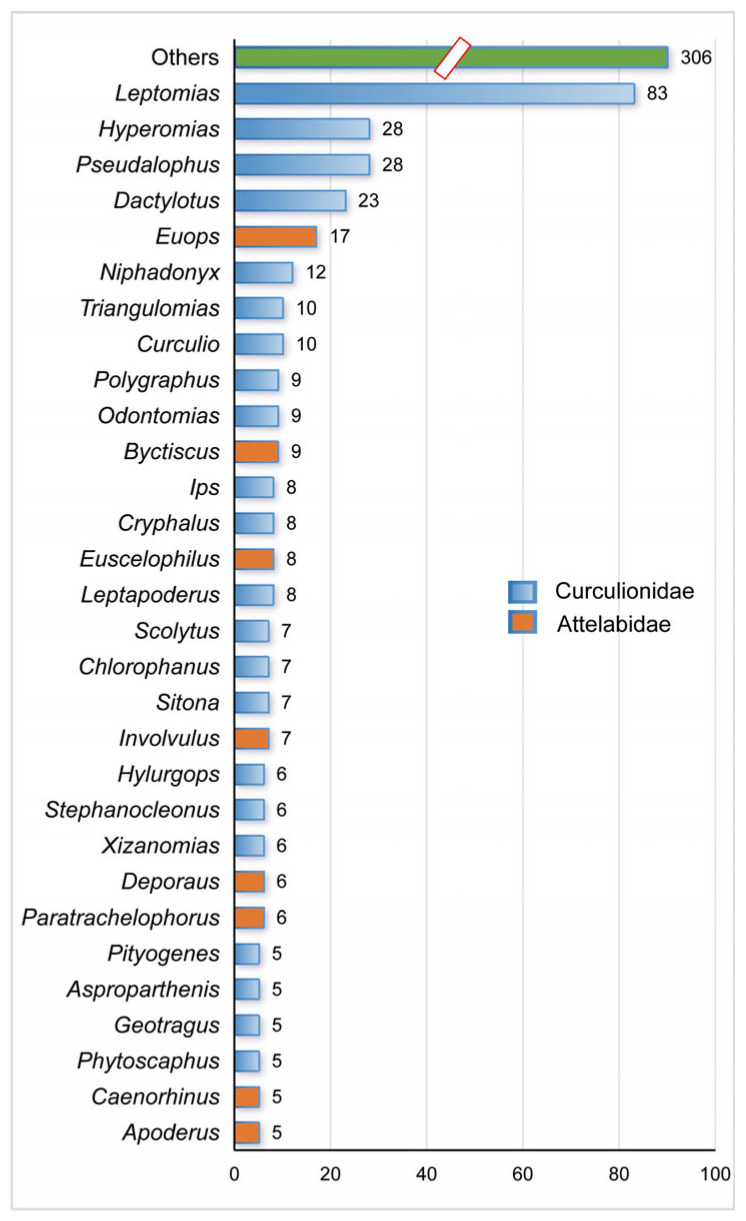
The genera composition of the Curculionoidea in the QXP. The numbers represent number of species they contain.

At the genus level, *Leptomias* was the most abundant genus, with 83 species (12.4%) (Figure 2), following by *Pseudalophus* (29 species, 4.3%), *Hyperomias* (28 species, 4.2%) and *Dactylotus* (23 species, 3.4%). Each of these four genera contains more than 20 species. More than half of the weevil species were represented by 13.5% (30/223) of the genera, with each of these genera containing at least five species.

### 3.2. Spatial Patterns of Species Richness and Determinants

**Figure 3:**
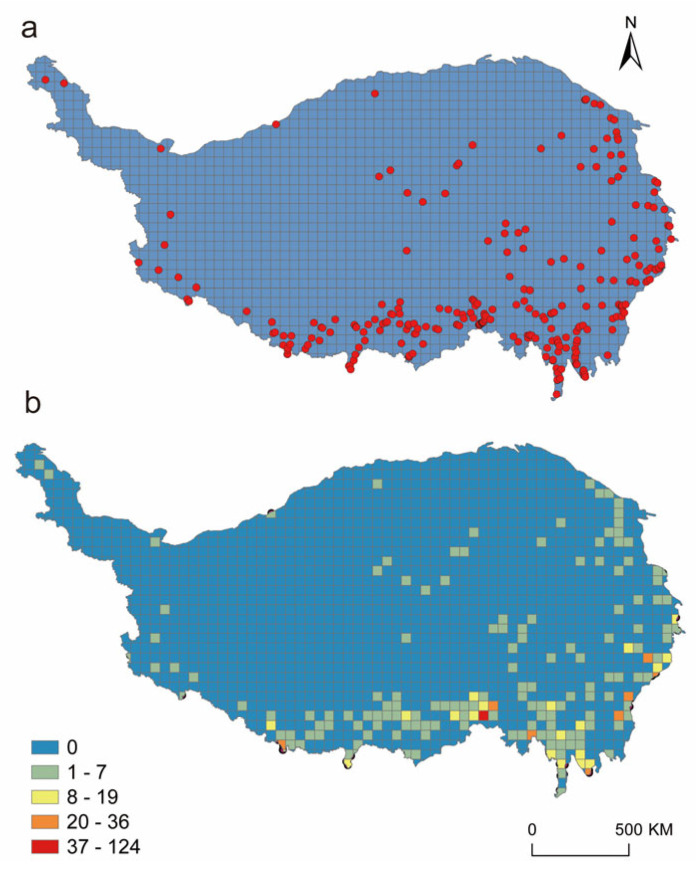
The distribution (**a**) and richness patterns (**b**) of Curculionoidea insects on the QXP.

The distribution of Curculionoidea from QXP was uneven, mainly concentrated in the eastern and southern of the plateau, while being scarce in the northern and central parts (Figure 3). Areas with high species diversity are concentrated in three regions: (1) northwestern Sichuan; (2) southeastern Xizang; (3) northwestern Yunnan. These regions are recognized as centers of species diversity for weevils. They also align closely with the two global biodiversity hotspots of South-Central China and Indo-Burma.

**Figure 4:**
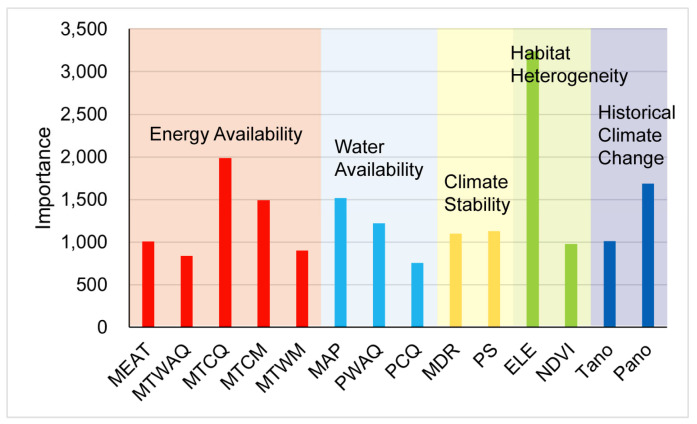
Results of Random Forest for the importance of all variables for Curculionoidea insects in the QXP. Importance values (y-axis) are represented by the increase in node purity. MEAT, mean annual temperature; MTWAQ, mean temperature of warmest quarter; MTCQ, mean temperature of coldest quarter; MTCM, minimum temperature of coldest month; MTWM, maximum temperature of warmest month; MAP, mean annual precipitation; PWAQ, precipitation of warmest quarter; PCQ, precipitation of coldest quarter; MDR, mean diurnal range; PS, precipitation seasonality; ELE, elevation range; NDVI, normalized difference vegetation index; Tano, MAT anomaly; Pano, MAP anomaly.

Univariate GLMs showed that the dominant factors for species richness were habitat heterogeneity (such as ELE and NDVI), water availability (such as MAP, PWAQ, and PCQ), energy availability (such as MEAT MTCP, and MTCM), contemporary climate stability (such as MDR), and historical climate change (such as Tano) (Table 1). Specifically, ELE was the strongest predictor, accounting for 30.25% (*p* < 0.001) of the explanatory power, followed by Tano (*p* < 0.001), MTCM (*p* < 0.001), MTCQ (*p* < 0.001), NDVI (*p* < 0.05), and so on. The results of Random Forest were with those of GLMs (Figure 4), with some differences in the order of relative importance. Variables ELE is the most significant. In addition, the MEAT + MAP + MDR + PS + EE + Tano + Pano model was selected as the optimal model by the multivariate approache. Broadly consistent with the above results, ELE remains a strong explanatory variable (Figures S6 and S7).

### 3.3. Species Delimitation and Cryptic Species Diversity

**Figure 5:**
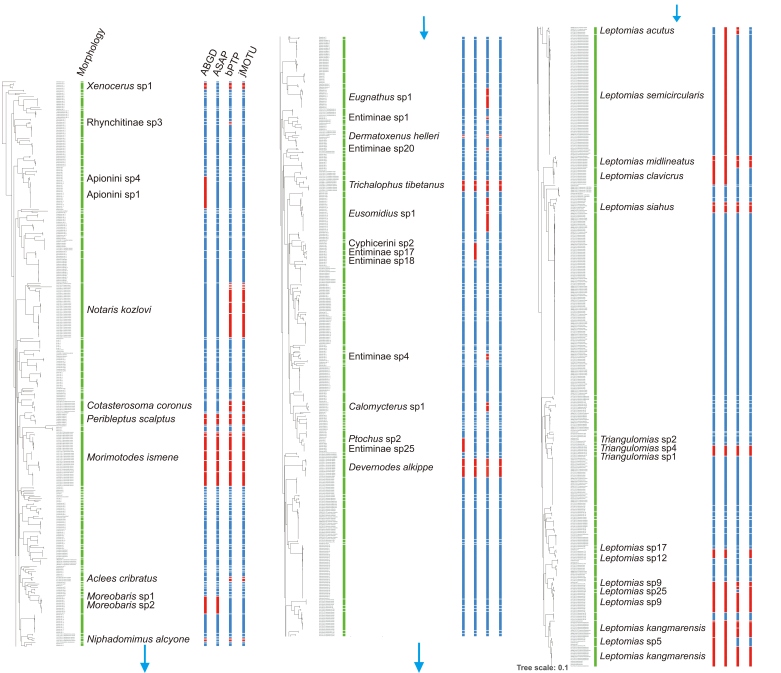
Species delimitation of Curculionoidea insects in the QTP based on morphology, automatic barcode gap discovery (ABGD), assemble species by automatic partitioning (ASAP), Poisson tree process (bPTP), jMOTU, are shown by bars next to species names. The red band denotes a mismatch between the molecular and morphological classifications.

The complete dataset encompassed 1147 COI-5′ barcode sequences from 217 morphological species within 4 families and 14 subfamilies. Based four methods of species delimitation, the number of molecule operational taxonomic units (MOTUs) ranged from 220 to 251 (Figure 5, Table S2). The ASAP analyses yielded a relatively conservative result of 220 MOTUs, with taxonomic concordance observed for 195 MOTUs when compared to the results obtained through morphological identification. ABGD methods recognized a total of 228 MOTUs, out of which 194 corresponded to the identified morphospecies. The jMOTU analyses identified a total of 240 MOTUs, with approximately 200 MOTUs showing consistent classification compared with the morphological classification. The bPTP method yielded the largest number of MOTU, with 193 being consistent with morphological results.

The results of our study have uncovered certain inconsistencies between molecular and morphospecies. Specifically, based on three or more species delimitation methods, a total of 11 morphospecies were each assigned to more than one MOTU (Figure 5, Table 2). With large intra-GD, there may be cryptic species within these species. *Notaris kozlovi* exemplifies pronounced cryptic diversity, exhibiting the highest intra-GD (29.27%) among studied taxa, with 34 sequences delineated into 7 MOTUs. This may exceptional phylogeographic structure underscores significant undocumented lineage differentiation within the species complex. Notably, with the exception of *Peribleptus scalptus*, *Xenocerus* sp1, and *Dermatoxenus helleri*, all species are endemic to the QXP and possess underdeveloped wings, rendering them incapable of flight.

## 4. Discussion

### 4.1. The Spatial Patterns and Drivers of Species Richness

Preliminary studies on the diversity patterns of various taxa within the QXP indicate that the majority of species are concentrated along its margins [43,57,58]. Similarly, the species diversity center of QXP weevils is located in southeastern Xizang, northwestern Sichuan, and northwestern Yunnan. Habitat heterogeneity, especially ELE, is the most important factor in explaining the distribution pattern of insect diversity. ELE, elevation range, was defined as the difference between the maximum elevation and the minimum elevation inside each grid cell. It may have affected biodiversity patterns in two ways.

First of all, the topographical barriers created by the uplift of the QXP may driven species differentiation and adaptive radiation for weevils. In terms of distribution, the four most diverse genera of weevils from QXP, *Leptomias*, *Pseudalophus*, *Hyperomias*, and *Dactylotus*, are all endemic to the QXP. Given that their elytra are fused, rendering them flightless, their dispersal capabilities are inherently limited, making them more vulnerable to geographical barriers. Taking *Leptomias* species as an example, most species (57 species, 68.7%) were distributed in the Hengduan Mountains and southeastern Xizang. This distribution pattern might be attributed to the fact that the Hengduan Mountains [59] and the Himalayas [60] are characterized by numerous peaks and valleys, which create a pronounced isolating effect conducive to the emergence of unique speciation events. In contrast, only 27 species of *Leptomias* are found within the plateau’s interior, which is less than half of those in the aforementioned regions. This could be due to the relatively flat terrain at the plateau’s core, resulting in a weaker isolating effect. *Leptomias semicircularis* Chao stands out as the most widely distributed species across the QXP, with its range extending to the middle and lower reaches of the Yarlung Zangbo River. Apart from the weak topographic isolation, a significant factor contributing to its broad distribution is its ability to disperse along the valley. Furthermore, only four species of *Leptomias* are present in North and Northeast China. Among them, *Leptomias schoenherri* Faust exhibits the broadest distribution within this genus, spanning North and Northeast China. The extensive distribution of this species may be linked to the relatively flat terrain in these areas [61], which presents far fewer topographic impediments during dispersal compared to the challenges posed by the QXP and its surrounding mountain ranges.

Molecular data appears to corroborate this perspective. Among the 11 species potentially harboring cryptic diversity, eight are flightless, a trait that hinders their ability to disperse across mountainous terrain. Consider, for instance, *Trichalophus tibetanus*, the sequences analyzed in this study originated from two distinct regions within the Hengduan Mountains: the central Gongga Mountain and the northern Songpan County (Figure S8). The significant intraspecific genetic divergence observed (14.49%) could be attributed to the geographical separation of their populations by mountain ranges, which impedes gene flow between them. Additional research has indicated that the geographical isolation resulting from the uplift of the Hengduan Mountains has played a role in the population differentiation and speciation of weevils [62,63,64,65,66,67]. Additionally, research into the phylogenetic and historical biogeographical patterns of *Gnaptorina* (Coleoptera: Tenebrionidae) species inhabiting the QXP has revealed that the ridges and valleys sculpted by the plateau’s uplift have created significant impediments to species dispersal. This has resulted in the establishment of numerous isolated habitats, which in turn has fostered the allopatric diversification of *Gnaptorina* species [15]. The *Gnaptorina* species is centered on the QXP, where it lacks hind wings and exhibits limited dispersal capabilities. It typically resides under high-altitude rocks, and its dispersal abilities and biological traits closely resemble those of the plateau’s endemic weevils. In summary, the geographical barriers erected by the uplift of the QXP have been instrumental in driving the divergence and diversification of weevil species.

And the emergence of new habitats may also play an important role. With the uplift of the mountains, the decrease in air temperature would shift the forest line, thus increasing different habitats such as shrubs, grasslands and deserts [60], especially in the interlocking ridges and valleys of the Hengduan Mountains [21,68]. The emergence of these new habitats presents increased ecological opportunities for species, and the adaptation to these novel ecological niches can drive species differentiation and speciation, a pattern observed across plants [69,70,71], amphibious reptiles [72,73], and insects [10,74,75,76]. A positive correlation has been established between the richness of plant lineages and the speciation rates of insect herbivores [77], which means that areas with higher plant species richness tend to have higher speciation rates and likely harbor more species. The ability to adapt to niche food resources is a key driver of species diversity [74,75,76].

### 4.2. Enlightenment of Cryptic Diversity of Weevils in the QXP

Regarding biogeographical distribution, weevils exhibit a cosmopolitan distribution across nearly all terrestrial ecosystems [28]. This remarkable adaptability is particularly evident on the QXP, where they occupy elevational gradients spanning 600–5600 m above sea level (as exemplified by *Leptomias huangi* Chao at 670 m, *Xizanomias altus* Chao and *Hyperomias marginatus* Aslam at 5600 m). Their species diversity on the QXP demonstrates striking ecological significance: current records approximate the avian diversity of this region, surpass many vertebrate groups in species richness, and account for approximately 50% of documented Hemiptera species. These combined attributes—broad environmental tolerance, exceptional elevational range, and disproportionate diversity—position weevils as an exemplary model system for investigating mechanisms of biodiversity origination and maintenance in extreme high-altitude ecosystems.

Our investigation of weevil diversity on the QXP provides critical insights into contemporary challenges of species delimitation. A representative case involves *Notaris kozlovi*, for which all BOLD database sequences originate from Grebennikov’s submissions. Our analysis of 34 specimens across seven genetic loci from Yunnan and Sichuan mountainous regions revealed extraordinary intra-GD, with maximum COI genetic distances reaching 29.27%—a value exceeding conventional thresholds for species-level differentiation in Coleoptera [78,79]. This molecular evidence strongly suggests the presence of at least seven putative cryptic species. Notably, Grebennikov’s [66] original description neither conducted morphological comparisons with type specimens nor performed molecular validation, despite the absence of diagnostic characters in external morphology and genitalia across populations. Phylogenetic reconstruction demonstrates geographically structured COI clades that correlate with sampling localities, yet all specimens were taxonomically assigned to *N. kozlovi* solely based on superficial morphological similarity. Parallel patterns emerge in the *Morimotodes ismene* complex, reinforcing the prevalence of cryptic diversity in QXP weevils. In some cases, morphological approaches and limited gene segments alone may not be sufficient to illustrate the validity of the species, and more molecular evidence (mitochondrial genome or whole genome) and ecological evidence may be required.

The discovery of cryptic diversity underscores that there are still significant gaps in the discovery and description of biodiversity within the QXP region, highlighting the urgent need for increased attention and research focus on this area. In this study, a total of 11 species were classified into 33 distinct MOTUs, thereby enhancing the recorded biodiversity of the QXP. Furthermore, the first author combined COI barcode data with morphological analyses to determine the species-level status of 17 novel species. These species were assigned to four genera: *Pachynotus* (3 species), *Odontomias* (3 species), *Triangulomias* (2 species),and *Leptomias* (9 species). They are currently being formally described and prepared for publication in an academic journal. This finding further demonstrates the untapped potential for biodiversity discovery in the QXP region. Beyond the weevil study [80], the substantial cryptic diversity documented in other taxa—such as fish [81], snakes [82], and plants [83]—suggests that the QXP harbors unexpected levels of biodiversity that remain to be fully explored. These collective findings emphasize the importance of continued integrative taxonomic approaches to uncover hidden diversity in this ecologically significant region.

### 4.3. Potential Influence of Sampling Bias

It is important to acknowledge that sampling effort was not uniform across the QXP, which could have influenced the observed patterns of weevil diversity and distribution. Due to the scarcity of weevil records from the QXP in online databases, the distribution data in this study primarily come from specimen collection and field surveys conducted mainly during two periods: 1973–1980 and 2018–2022. Between 2018 and 2022 alone, 15 field expeditions were conducted, totaling over 230 field days and covering approximately 48,000 km across the QXP. Despite the vastness of the plateau, our surveys, spanning two generations of researchers, achieved substantial coverage by targeting undersampled areas. Specimens were even collected at an elevation of 5600 m on Mount Everest (e.g., *Xizanomias altus* Chao).

However, we must also acknowledge that variations in collection intensity resulted from several logistical and environmental constraints. These included difficult terrain, limited accessibility in remote regions (e.g., the Hoh Xil uninhabited zone), and varying permissions for scientific collection in different areas. Such sampling heterogeneity could affect estimates of species richness and composition, as well as the inferred importance of environmental drivers—particularly if well-sampled regions differ systematically from undersampled ones in terms of elevation, habitat type, or climate.

In this study, we have taken measures to mitigate the potential influence of uneven sampling where possible. For instance, overdispersion in the GLM was addressed by specifying family = “quasipoisson”. Additionally, a modified *t*-test was applied to assess the significance of each regression coefficient, thereby controlling for the effects of spatial autocorrelation in significance testing [44]. Furthermore, the key environmental predictors identified—including elevation range—are derived from spatially continuous datasets and are not directly dependent on local collection density. We advise that interpretations of diversity hotspots in areas with notably low collection effort be made with caution. We also suggest that future studies explicitly account for sampling completeness when modeling biodiversity patterns across heterogeneous landscapes.

## 5. Conclusions

In this study, the distribution patterns and determinants of Curculionoidea insect diversity on the QXP were systematically investigated for the first time. Additionally, the first DNA barcode database for weevils on the QXP was established, aiming to elucidate the cryptic diversity of certain species through molecular delimitation. Based on 671 species, the observed species richness exhibited a markedly uneven distribution, characterized by higher diversity in the eastern and southern peripheries of the plateau. Three distinct centers of diversity were identified: northwestern Sichuan, southeastern Xizang, and northwestern Yunnan. Elevation range (ELE) emerged as the primary determinant shaping the diversity patterns of Curculionoidea insects on the QXP. The DNA barcode database comprises 1147 COI sequences from 217 species. Notably, 11 species were classified into multiple MOTUs by three or four molecular species delineation methods, suggesting the presence of potential cryptic diversity. Our findings highlight that biodiversity in the QXP region still needs more attention.

## Figures and Tables

**Table 1:** The explanatory power (R^2^_adj_, %) of each environmental variable on the patterns of species richness based on the univariate general linear models (GLMs). Modified *t*-test was used to test the significance.

Variables		R^2^_adj_
Energy Availability	MEAT	20.00 ***
	MTWAQ	7.77 **
	MTCQ	24.98 ***
	MTCM	25.14 ***
	MTWM	1.66
Water Availability	MAP	21.29 ***
	PWAQ	17.61 ***
	PCQ	3.74 *
Contemporary Climate Stability	MDR	17.80 ***
	PS	0.31
Habitat Heterogeneity	ELE	30.25 ***
	NDVI	21.34 **
Historical Climate Change	Tano	25.78 ***
	Pano	−0.02

* *p* < 0.1; ** *p* < 0.05; *** *p* < 0.001. MEAT, mean annual temperature; MTWAQ, mean temperature of warmest quarter; MTCQ, mean temperature of coldest quarter; MTCM, minimum temperature of coldest month; MTWM, maximum temperature of warmest month; MAP, mean annual precipitation; PWAQ, precipitation of warmest quarter; PCQ, precipitation of coldest quarter; MDR, mean diurnal range; PS, precipitation seasonality; ELE, elevation range; NDVI, normalized difference vegetation index; Tano, MAT anomaly; Pano, MAP anomaly.

**Table 2:** Some morphospecies containing possible cryptic diversity indicated by different species delimitation methods (ABGD, ASAP, bPTP, jMOTU).

Species	Number of Sequence	MOTUS				The Max-Intra-GD
		ASAP	ABGD	bPTP	jMOTU	
*Devernodes alkippe*	12	2	2	2	2	8.68%
*Leptomias siahus*	7	2	2	2	2	6.07%
*Leptomias kangmarensis*	24	1	3	4	3	12.24%
*Leptomias midlineatus*	8	1	2	2	2	7.96%
*Morimotodes ismene*	34	5	6	6	6	18.32%
*Notaris kozlovi*	34	7	7	7	7	29.05%
*Peribleptus scalptus*	7	2	2	2	2	9.45%
*Trichalophus tibetanus*	7	2	2	2	2	14.49%
*Xenocerus* sp1	4	1	2	2	2	7.76%
*Dermatoxenus helleri*	2	2	2	2	2	13.20%
*Niphadomimus alcyone*	2	1	2	2	2	6.23%

## Data Availability

The raw data supporting the conclusions of this article will be made available by the authors on request.

## Supplementary Materials

The following supporting information can be downloaded at: https://www.mdpi.com/article/10.3390/insects17010120/s1. References [84,85,86] are cited in the Supplementary Materials.
