# Supplementary material for: Unveiling Weevil Diversity Drivers and Cryptic Species on the Qinghai–Xizang Plateau
Source: Insects. 2026 Jan 21;17(1):120. doi: 10.3390/insects17010120 (PMC12841820; doi:10.3390/insects17010120)
Supplement: Supplementary file 1 [file insects-17-00120-s001.zip › insects-4037756-supplementary.pdf]

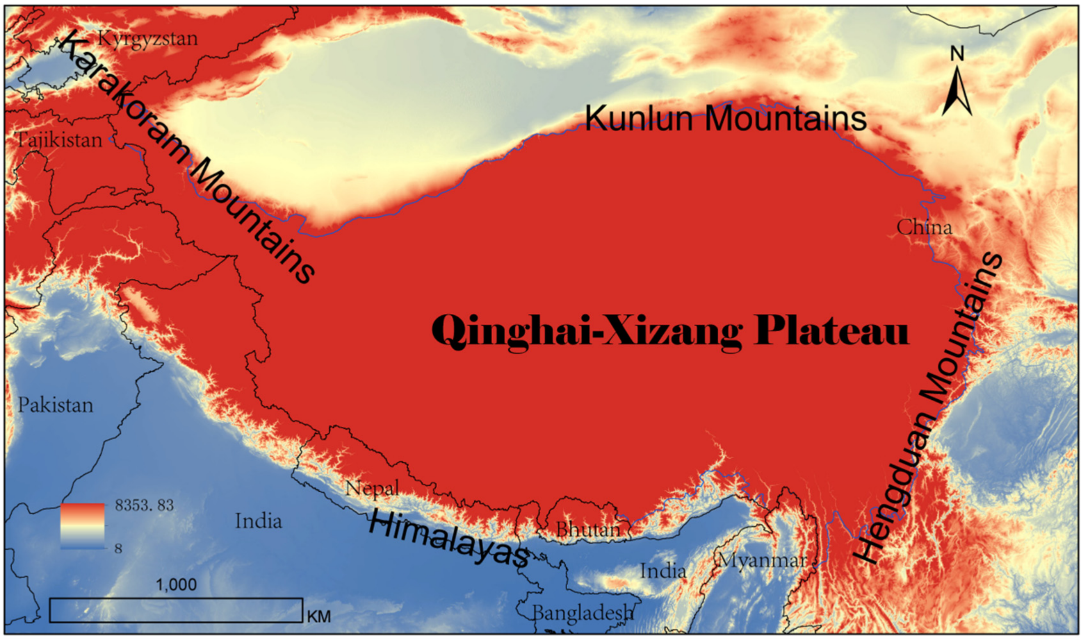

Fig. S1. Study area overview.

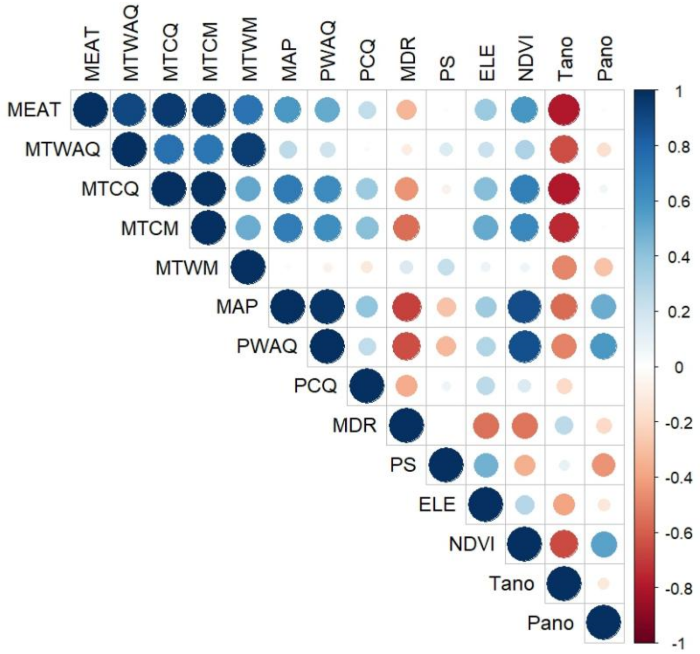

Fig. S2. The variable correlations of 14 environmental factors. MEAT, mean annual temperature; MTWAQ, mean temperature of warmest quarter; MTCQ, mean temperature of coldest quarter; MTCM, minimum temperature of coldest month; MTWM, maximum temperature of warmest month; MAP, mean annual precipitation; PWAQ, precipitation of warmest quarter; PCQ, precipitation of coldest quarter; MDR, mean diurnal range; PS, precipitation seasonality; ELE, elevation range; NDVI, normalized difference vegetation index; Tano, MAT anomaly; Pano, MAP anomaly.

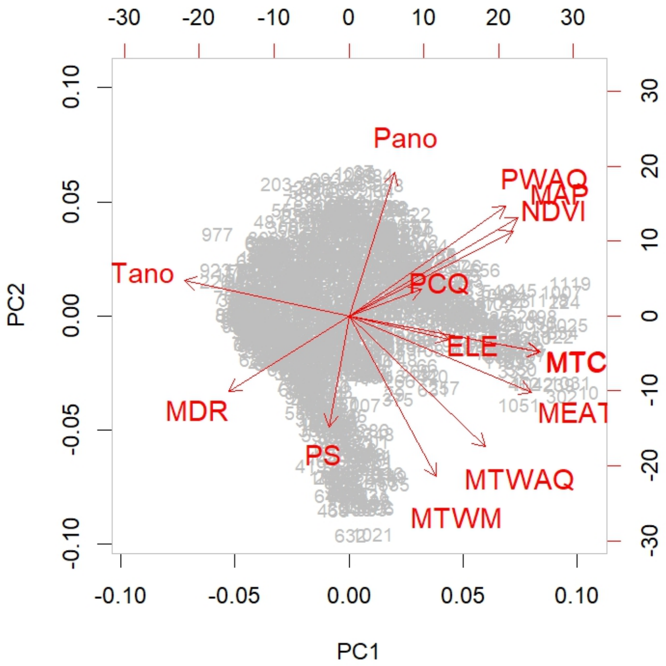

Fig. S3. The PCA analysis of 14 environmental factors. MEAT, mean annual temperature; MTWAQ, mean temperature of warmest quarter; MTCQ, mean temperature of coldest quarter; MTCM, minimum temperature of coldest month; MTWM, maximum temperature of warmest month; MAP, mean annual precipitation; PWAQ, precipitation of warmest quarter; PCQ, precipitation of coldest quarter; MDR, mean diurnal range; PS, precipitation seasonality; ELE, elevation range; NDVI, normalized difference vegetation index; Tano, MAT anomaly; Pano, MAP anomaly.

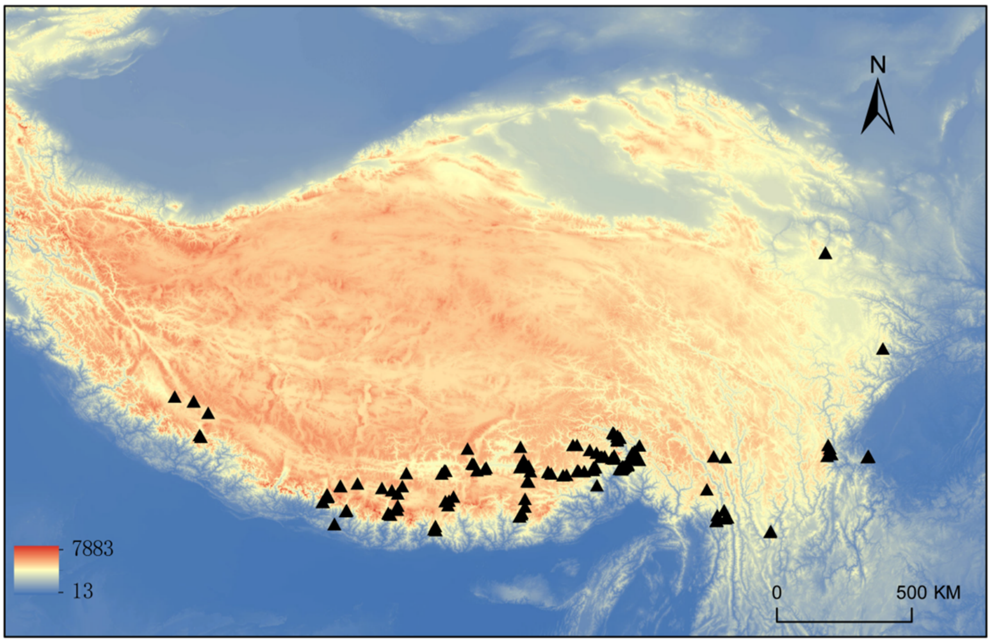

Fig. S4. Sampling point for COI.

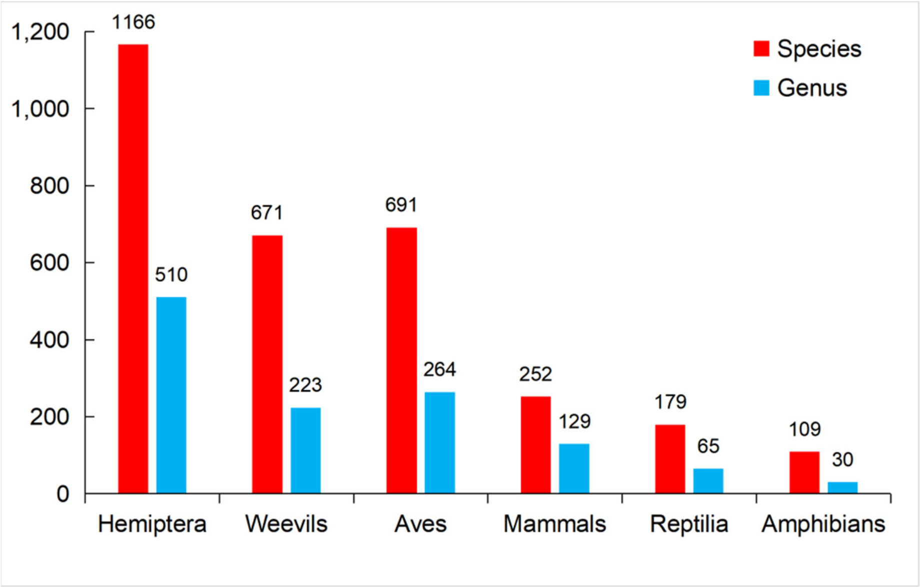

Fig. S5. Species diversity on the QXP.

We also collected data on Hemiptera insects (Li et al., 2019) amphibians (The Database of Chinese amphibians https://www.amphibiachina.org/distribution), reptiles (Guo and Che, 2024; The Reptile Database: https://reptile-database.reptarium.cz/), birds (Liu et al., 2013) and mammals (Wei et al., 2022) on the QTP to compare their diversity with that of weevils.

References

Guo, P., Che, J., 2024. Snakes in Qinghai-Xizang Plateau. Beijing, Science Press.

Li, J., Liu, H., Wu, Y., Zeng, L. and Huang, X. 2019. Spatial patterns and determinants of the diversity of Hemipteran insects in the Qinghai-Tibetan Plateau. Front. Ecol Evol. 7: 1–10.

Liu, Y., Bao, X. and Liao, J. 2013. The classification and distribution of the birds in Qingzang Plateau. Beijing, Science Press.

Wei, F., Yang, Q., Wu, Y., Jiang, X. and Liu, S. 2022. Taxonomy and distribution of mammals in China. Beijing, Science Press.

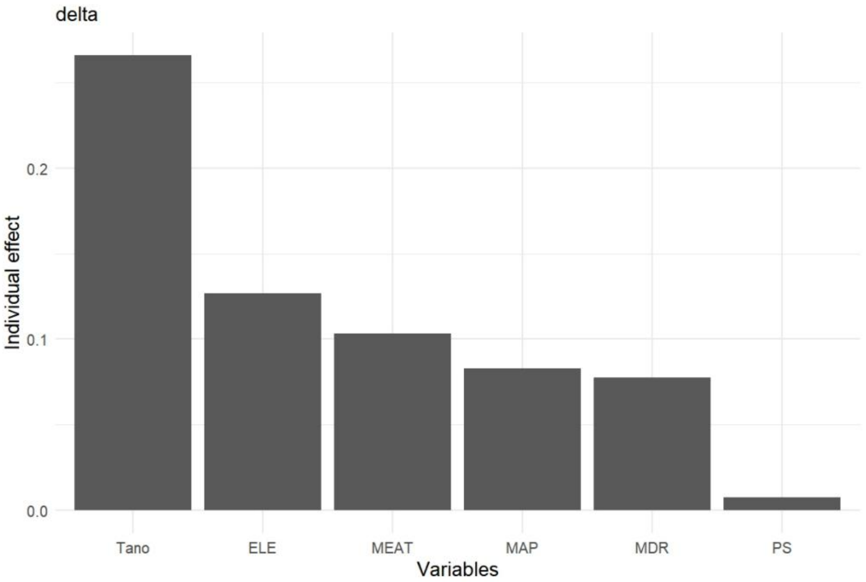

Fig. S6. Shared R^2 using glmm.hp. MEAT, mean annual temperature; MAP, mean annual precipitation; MDR, mean diurnal range; PS, precipitation seasonality; ELE, elevation range; Tano, MAT anomaly.

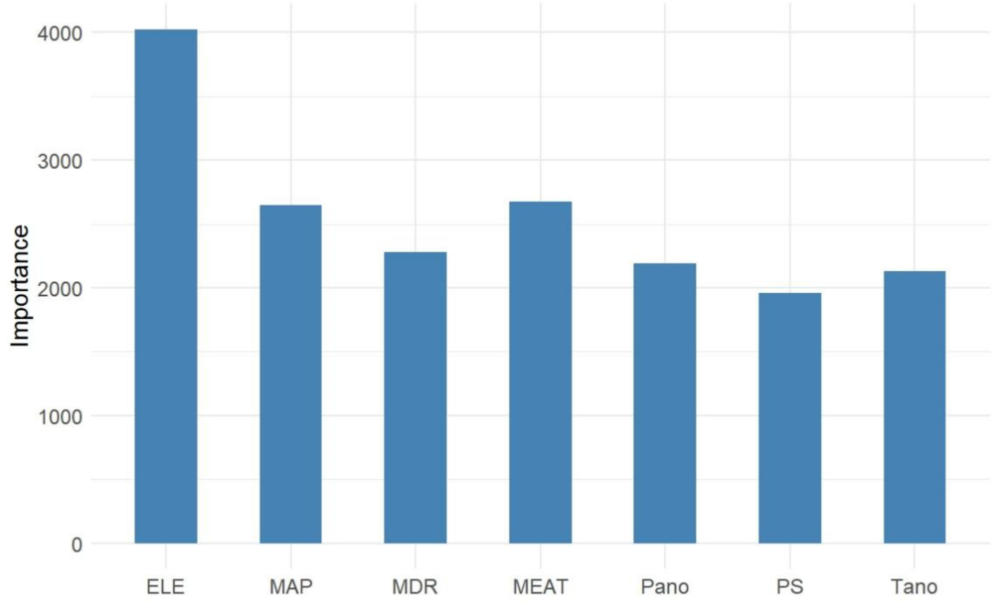

Fig. S7. Results of Random Forest for the importance of multivariate analyses. MEAT, mean annual temperature; MAP, mean annual precipitation; MDR, mean diurnal range; PS, precipitation seasonality; ELE, elevation range; Tano, MAT anomaly.

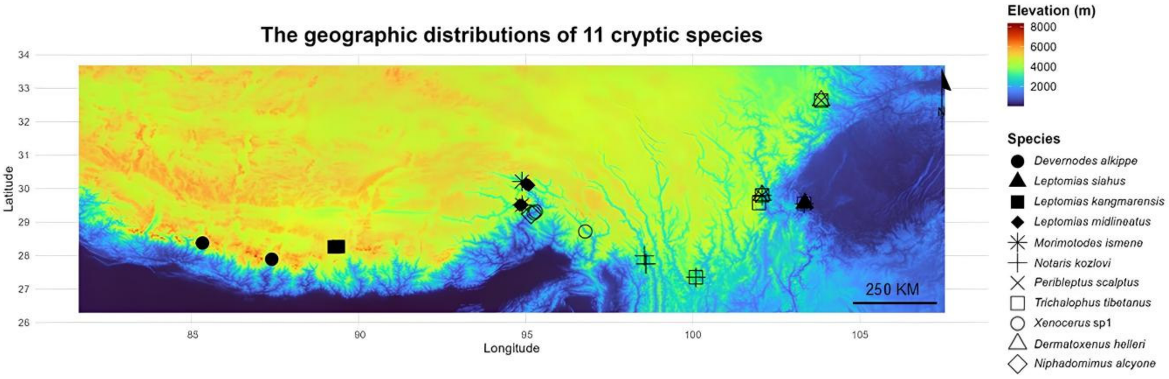

Fig. S8. The geographic distributions of 11 cryptic species.

Tab. S1. Specimen information.

Scientific name	Collection site	Latitude	Longitude	Collection time	Collector
Aclees cribratus	Gongshan County, Nujiang Prefecture, Yunnan	27.68	98.30	2021.5.31	Jinliang Ren
Anthribinae sp1	Motuo County, Linzhi City, Xizang	29.19	95.15	2019.8.11	Zhuo Ma
Anthribinae sp2	Bomi County, Linzhi City, Xizang	30.15	95.00	2019.7.20	Run Zhou
Apionini sp1	Motuo County, Linzhi City, Xizang	29.30	95.35	2019.7.28	Run Zhou
Apionini sp2	Motuo County, Linzhi City, Xizang	29.25	95.18	2018.8.22	Run Zhou
Apionini sp3	Motuo County, Linzhi City, Xizang	29.33	95.35	2018.8.20	Run Zhou
Apionini sp4	Gongshan County, Nujiang Prefecture, Yunnan	27.68	98.31	2019.8.23	Run Zhou
Apionini sp5	Fugong County, Lushui City, Yunnan	26.43	98.86	2021.5.28	Jinliang Ren
Apionini sp6	Jilong County, Rikaze City, Xizang	28.23	85.21	2019.8.6	Xiaoliang Yang
Apionini sp7	Jilong County, Rikaze City, Xizang	28.24	85.20	2019.8.9	Xiaoliang Yang
Attelabinae sp1	Nielamu County, Rikaze City, Xizang	27.97	85.97	2019.8.1	Xiaoliang Yang
Attelabinae sp2	Motuo County, Linzhi City, Xizang	29.19	95.15	2019.8.11	Zhuo Ma
Attelabinae sp3	Dingjie County, Rikaze City, Xizang	27.91	87.38	2021.7.24	Jinliang Ren
Attelabinae sp4	Jilong County, Rikaze City, Xizang	28.24	85.20	2019.8.9	Xiaoliang Yang
Baridini sp1	Gongshan County, Nujiang Prefecture, Yunnan	27.69	98.31	2021.5.30	Jinliang Ren
Baryrhynchus sp1	Motuo County, Linzhi City, Xizang	29.19	95.15	2019.8.11	Zhuo Ma
Baryrhynchus sp2	Motuo County, Linzhi City, Xizang	29.23	95.15	2021.6.12	Jinliang Ren
Calomycterus sp1	Geer County, Linzhi Region, Xizang	31.14	80.90	2021.8.9	Jinliang Ren
Cionus sp1	Bailang County, Rikaze City, Xizang	29.06	89.16	2019.7.26	Xiaoliang Yang
Cionus sp2	Dingjie County, Rikaze City, Xizang	27.89	87.40	2021.7.24	Jinliang Ren
Cionus sp3	Milin County, Linzhi City, Xizang	29.13	93.72	2022.7.22	Jinliang Ren
Cleonini sp1	Motuo County, Linzhi City, Xizang	29.19	95.08	2019.8.7	Run Zhou
Conoderinae sp1	Gongshan County, Nujiang Prefecture, Yunnan	27.69	98.31	2021.5.31	Jinliang Ren
Conoderinae sp10	Nielamu County, Rikaze City, Xizang	27.97	85.97	2019.8.1	Xiaoliang Yang

Conoderinae sp11	Nielamu County, Rikaze City, Xizang	27.97	85.97	2019.8.1	Xiaoliang Yang
Conoderinae sp12	Nielamu County, Rikaze City, Xizang	27.97	85.97	2019.8.1	Xiaoliang Yang
Conoderinae sp13	Nielamu County, Rikaze City, Xizang	27.58	85.58	2019.8.2	Xiaoliang Yang
Conoderinae sp14	Nielamu County, Rikaze City, Xizang	27.58	85.58	2019.8.2	Xiaoliang Yang
Conoderinae sp15	Nielamu County, Rikaze City, Xizang	27.98	85.98	2019.7.30	Xiaoliang Yang
Conoderinae sp16	Nielamu County, Rikaze City, Xizang	27.97	85.97	2019.8.1	Xiaoliang Yang
Conoderinae sp17	Motuo County, Linzhi City, Xizang	29.30	95.35	2019.7.28	Run Zhou
Conoderinae sp18	Motuo County, Linzhi City, Xizang	29.22	95.13	2019.7.30	Run Zhou
Conoderinae sp19	Motuo County, Linzhi City, Xizang	29.26	95.21	2022.7.18	Jinliang Ren
Conoderinae sp2	Gongshan County, Nujiang Prefecture, Yunnan	27.68	98.31	2019.8.23	Run Zhou
Conoderinae sp3	Jilong County, Rikaze City, Xizang	28.24	85.20	2019.8.9	Xiaoliang Yang
Conoderinae sp4	Motuo County, Linzhi City, Xizang	29.24	95.17	2019.8.6	Run Zhou
Conoderinae sp5	Nielamu County, Rikaze City, Xizang	27.58	85.58	2019.8.2	Xiaoliang Yang
Conoderinae sp6	Chayu County, Linzhi City, Xizang	28.72	96.78	2021.6.6	Jinliang Ren
Conoderinae sp7	Nielamu County, Rikaze City, Xizang	27.98	85.98	2019.7.3	Xiaoliang Yang
Conoderinae sp8	Nielamu County, Rikaze City, Xizang	27.97	85.97	2019.8.1	Xiaoliang Yang
Conoderinae sp9	Chayu County, Linzhi City, Xizang	28.49	97.02	2021.6.9	Jinliang Ren
Cosmopolites sordidus	Motuo County, Linzhi City, Xizang	29.35	95.34	2019.7.28	Zhuo Ma
Crypturgus sp1	Yadong County, Rikaze City, Xizang	27.43	88.93	2018.8.7	Run Zhou
Curculio sp1	Jilong County, Rikaze City, Xizang	28.38	85.35	2021.8.4	Jinliang Ren
Curculioninae sp1	Mangkang County, Changdu City, Xizang	29.56	98.20	2022.7.27	Jinliang Ren
Cyphicerini sp1	Naidong County, Shannan City, Xizang	29.45	91.88	2021.7.11	Jinliang Ren
Cyphicerini sp2	Milin County, Linzhi City, Xizang	29.50	94.90	2021.7.5	Jinliang Ren
Cyrtepistomus sp1	Yadong County, Rikaze City, Xizang	27.52	88.94	2021.7.22	Jinliang Ren
Dactylotinomorphus sp1	Milin County, Linzhi City, Xizang	29.51	94.82	2021.7.5	Jinliang Ren
Dactylotus sp1	Bomi County, Linzhi City, Xizang	30.10	95.09	2019.6.9	Zailin Wang

Dactylotus sp2	Nielamu County, Rikaze City, Xizang	27.58	85.58	2019.8.2	Xiaoliang Yang
Dermatoxenus helleri	Jilong County, Rikaze City, Xizang	28.37	85.33	2018.8.2	Run Zhou
Dryocoetes sp1	Motuo County, Linzhi City, Xizang	28.60	97.98	2019.8.18	Run Zhou
Dryophthorinae sp1	Motuo County, Linzhi City, Xizang	29.35	95.34	2019.7.28	Zhuo Ma
Dryophthorinae sp2	Motuo County, Linzhi City, Xizang	29.23	95.14	2019.8.5	Run Zhou
Dryophthorinae sp3	Motuo County, Linzhi City, Xizang	29.35	95.34	2019.7.28	Zhuo Ma
Dryophthorinae sp4	Motuo County, Linzhi City, Xizang	29.35	95.34	2019.7.28	Zhuo Ma
Dryophthorini sp1	Linzhi City, Xizang	29.56	94.58	2019.7.18	Run Zhou
Dryophthorini sp2	Linzhi City, Xizang	30.05	95.01	2019.7.21	Run Zhou
Dryophthorini sp3	Motuo County, Linzhi City, Xizang	29.70	95.56	2019.7.25	Run Zhou
Entiminae sp1	Jilong County, Rikaze City, Xizang	28.24	85.20	2019.8.9	Xiaoliang Yang
Entiminae sp10	Yadong County, Rikaze City, Xizang	27.52	88.94	2021.7.22	Jinliang Ren
Entiminae sp11	Jilong County, Rikaze City, Xizang	28.38	85.35	2021.8.4	Jinliang Ren
Entiminae sp12	Jilong County, Rikaze City, Xizang	28.37	85.33	2018.8.2	Run Zhou
Entiminae sp13	Gongshan County, Nujiang Prefecture, Yunnan	27.68	98.31	2019.8.21	Run Zhou
Entiminae sp14	Milin County, Linzhi City, Xizang	29.59	94.90	2021.7.4	Jinliang Ren
Entiminae sp15	Renbu County, Rikaze City, Xizang	29.35	90.17	2021.6.27	Jinliang Ren
Entiminae sp16	Qushui County, Lasa City, Xizang	29.16	90.33	2019.7.24	Xiaoliang Yang
Entiminae sp17	Milin County, Linzhi City, Xizang	29.25	94.27	2021.7.6	Jinliang Ren
Entiminae sp18	Milin County, Linzhi City, Xizang	29.23	94.21	2021.7.6	Jinliang Ren
Entiminae sp19	Milin County, Linzhi City, Xizang	29.16	94.20	2021.7.7	Jinliang Ren
Entiminae sp2	Milin County, Linzhi City, Xizang	29.25	94.27	2021.7.6	Jinliang Ren
Entiminae sp20	Luding County, Ganzi Prefecture, Sichuan	29.89	102.01	2011.6.4	Huakang Zhang
Entiminae sp21	Milin County, Linzhi City, Xizang	29.50	94.90	2021.7.5	Jinliang Ren
Entiminae sp22	Motuo County, Linzhi City, Xizang	29.30	95.35	2019.7.28	Run Zhou
Entiminae sp23	Gongshan County, Nujiang Prefecture, Yunnan	27.70	98.35	2021.5.30	Jinliang Ren

Entiminae sp24	Mangkang County, Changdu City, Xizang	29.54	98.59	2022.7.28	Jinliang Ren
Entiminae sp25	Lang County, Linzhi City, Xizang	29.02	93.32	2022.7.23	Jinliang Ren
Entiminae sp3	Milin County, Linzhi City, Xizang	29.51	94.82	2021.7.5	Jinliang Ren
Entiminae sp4	Motuo County, Linzhi City, Xizang	29.24	95.17	2019.8.6	Run Zhou
Entiminae sp5	Motuo County, Linzhi City, Xizang	29.66	95.49	2019.7.25	Run Zhou
Entiminae sp6	Dingjie County, Rikaze City, Xizang	27.91	87.38	2021.7.24	Jinliang Ren
Entiminae sp7	Gongshan County, Nujiang Prefecture, Yunnan	27.68	98.31	2019.8.21	Run Zhou
Entiminae sp8	Gongshan County, Nujiang Prefecture, Yunnan	27.68	98.31	2019.8.21	Run Zhou
Entiminae sp9	Nielamu County, Rikaze City, Xizang	27.97	85.97	2019.8.1	Xiaoliang Yang
Eucorynus sp1	Motuo County, Linzhi City, Xizang	29.23	95.14	2019.8.5	Run Zhou
Eugnathus distinctus	Dingjie County, Rikaze City, Xizang	27.91	87.38	2021.7.24	Jinliang Ren
Eugnathus sp1	Motuo County, Linzhi City, Xizang	29.30	95.35	2019.7.28	Run Zhou
Eugnathus sp2	Motuo County, Linzhi City, Xizang	29.23	95.18	2022.7.17	Jinliang Ren
Eusomidius sp1	Jilong County, Rikaze City, Xizang	28.23	85.21	2019.8.6	Xiaoliang Yang
Eusomidius sp2	Jilong County, Rikaze City, Xizang	28.37	85.33	2018.8.2	Run Zhou
Euwallacea interjectus	Motuo County, Linzhi City, Xizang	29.25	95.20	2019.8.8	Run Zhou
Gasteroclisus arcurostris	Motuo County, Linzhi City, Xizang	29.19	95.15	2019.8.11	Zhuo Ma
Geotragus himalayanus	Yadong County, Rikaze City, Xizang	27.43	88.90	2021.7.20	Jinliang Ren
Geotragus tuberculatus	Gongshan County, Nujiang Prefecture, Yunnan	27.68	98.31	2019.8.21	Run Zhou
Hylobius sp1	Motuo County, Linzhi City, Xizang	28.60	97.98	2019.8.18	Run Zhou
Hyperomias sp1	Qusong County, Shannan City, Xizang	28.83	92.05	2021.7.15	Jinliang Ren
Hyperomias sp2	Dingjie County, Rikaze City, Xizang	28.00	87.68	2021.7.27	Jinliang Ren
Ips sp1	Yadong County, Rikaze City, Xizang	27.43	88.93	2018.8.7	Run Zhou
Lagenolobus sp1	Chayu County, Linzhi City, Xizang	28.76	96.75	2021.6.6	Jinliang Ren
Leptomias acutus	Qusong County, Shannan City, Xizang	29.16	90.33	2019.7.24	Xiaoliang Yang
Leptomias clarus	Dingri County, Rikaze City, Xizang	28.57	87.49	2021.7.28	Jinliang Ren

Leptomias clavicrus	Milin County, Linzhi City, Xizang	29.51	94.89	2021.7.2	Jinliang Ren
Leptomias crinitarsus	Milin County, Linzhi City, Xizang	29.13	93.90	2021.6.2	Jinliang Ren
Leptomias depressus	Milin County, Linzhi City, Xizang	30.02	95.01	2019.7.22	Run Zhou
Leptomias erectus	Cuona County, Shannan City, Xizang	27.82	91.75	2021.7.12	Jinliang Ren
Leptomias hirsutus	Milin County, Linzhi City, Xizang	29.51	94.89	2021.7.2	Jinliang Ren
Leptomias huangi	Motuo County, Linzhi City, Xizang	29.23	95.15	2021.6.14	Jinliang Ren
Leptomias kangmarensis	Kangma County, Rikaze City, Xizang	28.27	89.39	2021.7.23	Jinliang Ren
Leptomias kingdonwardi	Bomi County, Linzhi City, Xizang	30.24	94.85	2019.9.1	Xiaoliang Yang
Leptomias lineatus	Gongbujiangda County, Linzhi City, Xizang	29.89	93.66	2022.7.25	Jinliang Ren
Leptomias mainlingensis	Milin County, Linzhi City, Xizang	29.23	94.21	2021.7.6	Jinliang Ren
Leptomias mangkamensis	Mangkang County, Changdu City, Xizang	29.56	98.20	2022.7.27	Jinliang Ren
Leptomias micans	Yadong County, Rikaze City, Xizang	27.43	88.91	2018.8.5	Run Zhou
Leptomias midlineatus	Bomi County, Linzhi City, Xizang	30.10	95.09	2019.6.9	Zailin Wang
Leptomias sagaensis	Geer County, Linzhi Region, Xizang	31.14	80.90	2021.8.9	Jinliang Ren
Leptomias semicircularis	Milin County, Linzhi City, Xizang	29.15	93.68	2022.7.23	Jinliang Ren
Leptomias siahus	Bomi County, Linzhi City, Xizang	30.21	94.89	2018.8.25	Run Zhou
Leptomias sp1	Sajia County, Rikaze City, Xizang	28.69	87.85	2021.6.22	Jinliang Ren
Leptomias sp10	Kangma County, Rikaze City, Xizang	28.38	89.54	2021.7.19	Jinliang Ren
Leptomias sp11	Kangma County, Rikaze City, Xizang	28.26	89.27	2021.7.23	Jinliang Ren
Leptomias sp12	Jiacha County, Shannan City, Xizang	29.11	92.69	2021.7.9	Jinliang Ren
Leptomias sp13	Nielamu County, Rikaze City, Xizang	28.70	85.79	2021.7.29	Jinliang Ren
Leptomias sp14	Naidong County, Shannan City, Xizang	29.45	91.88	2021.7.11	Jinliang Ren
Leptomias sp15	Linzhi City, Xizang	28.72	94.32	2019.7.30	Wei Wang
Leptomias sp16	Lang County, Linzhi City, Xizang	29.07	92.80	2018.8.16	Run Zhou
Leptomias sp17	Qusong County, Shannan City, Xizang	29.13	92.11	2019.8.13	Wei Wang
Leptomias sp18	Linzhi City, Xizang	29.75	94.08	2019.8.13	Wei Wang

Leptomias sp19	Nielamu County, Rikaze City, Xizang	27.59	85.59	2019.8.4	Xiaoliang Yang
Leptomias sp2	Nimu County, Lasa City, Xizang	29.33	90.23	2021.6.22	Jinliang Ren
Leptomias sp20	Naidong County, Shannan City, Xizang	29.45	91.88	2021.7.10	Jinliang Ren
Leptomias sp21	Yadong County, Rikaze City, Xizang	28.14	89.37	2018.8.8	Chengjing Liu
Leptomias sp22	Motuo County, Linzhi City, Xizang	29.26	95.21	2020.9.17	Xiaoliang Yang
Leptomias sp23	Saga County, Rikaze City, Xizang	28.24	85.20	2019.8.10	Xiaoliang Yang
Leptomias sp24	Milin County, Linzhi City, Xizang	29.25	94.27	2021.7.6	Jinliang Ren
Leptomias sp25	Milin County, Linzhi City, Xizang	29.15	93.67	2021.7.8	Jinliang Ren
Leptomias sp26	Motuo County, Linzhi City, Xizang	29.32	95.32	2021.6.8	Jinliang Ren
Leptomias sp27	Bailang County, Rikaze City, Xizang	29.15	89.29	2021.7.18	Jinliang Ren
Leptomias sp28	Lang County, Linzhi City, Xizang	29.02	93.19	2022.7.23	Jinliang Ren
Leptomias sp29	Naidong County, Shannan City, Xizang	28.84	92.00	2021.7.12	Jinliang Ren
Leptomias sp3	Jiacha County, Shannan City, Xizang	29.11	92.70	2022.7.24	Jinliang Ren
Leptomias sp30	Mozhugongka County, Lasa City, Xizang	29.84	91.77	2018.7.21	Runzhi Zhang
Leptomias sp31	Linzhi City, Xizang	29.55	94.49	2018.7.20	Runzhi Zhang
Leptomias sp4	Cuona County, Shannan City, Xizang	28.32	91.92	2021.7.14	Jinliang Ren
Leptomias sp5	Cuona County, Shannan City, Xizang	27.92	91.82	2021.7.12	Jinliang Ren
Leptomias sp6	Dingjie County, Rikaze City, Xizang	28.13	87.70	2021.7.23	Jinliang Ren
Leptomias sp7	Cuona County, Shannan City, Xizang	28.10	91.93	2018.8.15	Run Zhou
Leptomias sp8	Dingjie County, Rikaze City, Xizang	28.48	87.70	2021.6.26	Jinliang Ren
Leptomias sp9	Bailang County, Rikaze City, Xizang	29.15	89.29	2021.7.18	Jinliang Ren
Leptomias subundulans	Bomi County, Linzhi City, Xizang	30.15	95.01	2019.7.20	Run Zhou
Leptomias undulans	Chayu County, Linzhi City, Xizang	28.80	96.72	2021.6.6	Jinliang Ren
Leptomias viridicantis	Motuo County, Linzhi City, Xizang	29.23	95.18	2022.7.17	Jinliang Ren
Lixini sp1	Gongshan County, Nujiang Prefecture, Yunnan	27.68	98.31	2019.8.23	Run Zhou
Lixus sp1	Jilong County, Rikaze City, Xizang	28.23	85.21	2019.8.6	Xiaoliang Yang

Lixus sp2	Chayu County, Linzhi City, Xizang	28.76	96.75	2021.6.6	Jinliang Ren
Lixus sp3	Nielamu County, Rikaze City, Xizang	27.97	85.97	2019.8.1	Xiaoliang Yang
Lobotrachelus sp1	Cuona County, Shannan City, Xizang	29.29	91.82	2021.7.13	Jinliang Ren
Mecopini sp1	Motuo County, Linzhi City, Xizang	29.46	95.74	2019.8.15	Run Zhou
Merus flavosignatus	Gongshan County, Nujiang Prefecture, Yunnan	27.68	98.31	2019.8.23	Run Zhou
Merus sp1	Nielamu County, Rikaze City, Xizang	27.97	85.97	2019.8.1	Xiaoliang Yang
Molytinae sp1	Nielamu County, Rikaze City, Xizang	27.98	85.98	2019.7.30	Xiaoliang Yang
Molytinae sp2	Gongshan County, Nujiang Prefecture, Yunnan	27.68	98.31	2019.8.23	Run Zhou
Molytinae sp3	Motuo County, Linzhi City, Xizang	29.23	95.18	2021.6.11	Jinliang Ren
Molytinae sp4	Motuo County, Linzhi City, Xizang	29.66	95.49	2019.7.25	Run Zhou
Molytinae sp5	Motuo County, Linzhi City, Xizang	29.71	95.59	2019.7.24	Run Zhou
Molytinae sp6	Gongshan County, Nujiang Prefecture, Yunnan	27.69	98.31	2021.5.31	Jinliang Ren
Molytinae sp7	Gongshan County, Nujiang Prefecture, Yunnan	27.68	98.31	2019.8.23	Run Zhou
Molytinae sp8	Motuo County, Linzhi City, Xizang	29.32	95.32	2018.8.20	Run Zhou
Molytinae sp9	Motuo County, Linzhi City, Xizang	29.32	95.31	2019.8.14	Run Zhou
Moreobaris sp1	Nielamu County, Rikaze City, Xizang	27.98	85.98	2019.7.30	Xiaoliang Yang
Moreobaris sp2	Jilong County, Rikaze City, Xizang	28.23	85.21	2019.8.6	Xiaoliang Yang
Myllocerus viridis	Motuo County, Linzhi City, Xizang	29.24	95.19	2019.7.29	Run Zhou
Nedyus quadrimaculatus	Cuona County, Shannan City, Xizang	29.29	91.82	2021.7.13	Jinliang Ren
Niphades sp1	Motuo County, Linzhi City, Xizang	29.30	95.35	2019.7.28	Run Zhou
Niphades sp2	Motuo County, Linzhi City, Xizang	29.25	95.19	2019.7.31	Run Zhou
Niphades variegatus	Motuo County, Linzhi City, Xizang	29.71	95.59	2019.7.24	Run Zhou
Odoiporus longicollis	Motuo County, Linzhi City, Xizang	29.35	95.34	2019.7.28	Zhuo Ma
Odontomias crassus	Qusong County, Shannan City, Xizang	29.13	92.11	2019.8.13	Wei Wang
Odontomias latus	Nimu County, Lasa City, Xizang	29.33	90.23	2021.6.22	Jinliang Ren
Odontomias nigrolatus	Qushui County, Lasa City, Xizang	29.16	90.33	2019.7.24	Xiaoliang Yang

Odontomias sp1	Naidong County, Shannan City, Xizang	29.29	91.82	2021.7.10	Jinliang Ren
Odontomias sp2	Bailang County, Rikaze City, Xizang	29.15	89.29	2021.7.18	Jinliang Ren
Odontomias sp3	Bailang County, Rikaze City, Xizang	29.15	89.29	2021.7.18	Jinliang Ren
Odontomias sp4	Nimu County, Lasa City, Xizang	29.79	90.01	2021.7.16	Jinliang Ren
Odontomias sp5	Lazi County, Rikaze City, Xizang	29.08	87.98	2021.6.22	Jinliang Ren
Odontomias subparvilatus	Bailang County, Rikaze City, Xizang	29.06	89.16	2019.7.27	Xiaoliang Yang
Pachynotus lampoglobus	Pulan County, Linzhi Region, Xizang	30.18	81.11	2019.8.13	Xiaoliang Yang
Pachynotus pilosus	Qusong County, Shannan City, Xizang	28.83	92.05	2021.7.12	Jinliang Ren
Pachynotus sp1	Cuona County, Shannan City, Xizang	28.10	91.93	2018.8.15	Run Zhou
Paratrachelophorus sp1	Dingjie County, Rikaze City, Xizang	27.85	87.45	2021.6.25	Jinliang Ren
Peribleptus foveostriatus	Motuo County, Linzhi City, Xizang	29.24	95.17	2019.8.6	Run Zhou
Peribleptus scalptus	Chayu County, Linzhi City, Xizang	28.72	96.78	2021.6.6	Jinliang Ren
Phytoscaphus chloroticus	Motuo County, Linzhi City, Xizang	29.27	95.21	2020.9.8	Xiaoliang Yang
Phytoscaphus ciliatus	Motuo County, Linzhi City, Xizang	29.30	95.28	2020.9.12	Xiaoliang Yang
Phytoscaphus decorus	Motuo County, Linzhi City, Xizang	29.24	95.17	2019.8.6	Run Zhou
Phytoscaphus kaulbacki	Motuo County, Linzhi City, Xizang	29.23	95.16	2019.8.13	Run Zhou
Phytoscaphus lineatus	Motuo County, Linzhi City, Xizang	29.23	95.16	2019.8.13	Run Zhou
Phytoscaphus sp1	Motuo County, Linzhi City, Xizang	29.33	95.35	2018.8.20	Run Zhou
Phytoscaphus sp2	Chayu County, Linzhi City, Xizang	29.11	96.48	2021.6.5	Jinliang Ren
Phytoscaphus sp3	Nielamu County, Rikaze City, Xizang	27.97	85.97	2019.8.1	Xiaoliang Yang
Phytoscaphus sp4	Motuo County, Linzhi City, Xizang	29.24	95.19	2018.8.21	Run Zhou
Phytoscaphus sp5	Motuo County, Linzhi City, Xizang	29.23	95.18	2022.7.17	Jinliang Ren
Platymycteropsis sp1	Motuo County, Linzhi City, Xizang	29.23	95.18	2022.7.17	Jinliang Ren
Ptochus potanini	Nielamu County, Rikaze City, Xizang	28.70	85.79	2021.7.29	Jinliang Ren
Ptochus sp1	Bailang County, Rikaze City, Xizang	29.15	89.24	2021.7.17	Jinliang Ren
Ptochus sp2	Milin County, Linzhi City, Xizang	29.25	94.27	2021.7.6	Jinliang Ren

Rhynchitinae sp1	Cuona County, Shannan City, Xizang	27.87	91.81	2018.8.12	Run Zhou
Rhynchitinae sp2	Jilong County, Rikaze City, Xizang	28.24	85.20	2019.8.9	Xiaoliang Yang
Rhynchitinae sp3	Jilong County, Rikaze City, Xizang	28.24	85.20	2019.8.9	Xiaoliang Yang
Rhynchitinae sp4	Jilong County, Rikaze City, Xizang	28.38	85.35	2021.8.4	Jinliang Ren
Rhynchitinae sp5	Jilong County, Rikaze City, Xizang	28.24	85.20	2019.8.9	Xiaoliang Yang
Rhynchitinae sp6	Jilong County, Rikaze City, Xizang	28.24	85.20	2019.8.9	Xiaoliang Yang
Rhynchitinae sp7	Jilong County, Rikaze City, Xizang	28.45	85.35	2019.8.5	Xiaoliang Yang
Rhynchophorini sp1	Gongshan County, Nujiang Prefecture, Yunnan	27.84	98.32	2019.8.20	Run Zhou
Scolytinae sp1	Motuo County, Linzhi City, Xizang	29.25	95.20	2019.8.4	Run Zhou
Scolytinae sp2	Bomi County, Linzhi City, Xizang	30.24	94.86	2019.7.19	Run Zhou
Scolytinae sp3	Motuo County, Linzhi City, Xizang	29.22	95.18	2019.8.6	Run Zhou
Scolytinae sp4	Yadong County, Rikaze City, Xizang	27.43	88.93	2018.8.7	Run Zhou
Sitona cylindricollis	Bailang County, Rikaze City, Xizang	29.15	89.24	2021.7.17	Jinliang Ren
Sitona sp1	Motuo County, Linzhi City, Xizang	29.24	95.17	2019.8.6	Run Zhou
Sitona sp2	Nielamu County, Rikaze City, Xizang	27.99	85.99	2019.7.31	Xiaoliang Yang
Sitophilus oryzae	Motuo County, Linzhi City, Xizang	29.25	95.18	2018.8.22	Run Zhou
Sitophilus zeamais	Motuo County, Linzhi City, Xizang	29.25	95.18	2018.8.22	Run Zhou
Triangulomias sp1	Dingjie County, Rikaze City, Xizang	27.85	87.45	2021.7.26	Jinliang Ren
Triangulomias sp2	Milin County, Linzhi City, Xizang	29.15	93.68	2022.7.23	Jinliang Ren
Triangulomias sp3	Naidong County, Shannan City, Xizang	29.45	91.88	2021.7.11	Jinliang Ren
Triangulomias sp4	Dingri County, Rikaze City, Xizang	28.64	87.17	2021.7.28	Jinliang Ren
Triangulomias sp5	Kangma County, Rikaze City, Xizang	28.38	89.54	2021.7.19	Jinliang Ren
Trochorhopalus sp1	Motuo County, Linzhi City, Xizang	29.35	95.34	2019.7.28	Zhuo Ma
Tychius oriens	Nielamu County, Rikaze City, Xizang	28.78	86.35	2019.7.29	Xiaoliang Yang
Xenocerus sp1	Motuo County, Linzhi City, Xizang	29.23	95.16	2019.8.13	Run Zhou
Xizanomias sp1	Bailang County, Rikaze City, Xizang	29.15	89.24	2021.7.17	Jinliang Ren

Tab. S2. Species delimitation schemes of 1,147 COI sequences by ABGD, ASAP, bPTP and jMOTU.

Species name	Sequence number	ABGD	ASAP	bPTP	jMOTU
Anthribinae_sp1	1	Group-5	Group-5	Group-83	Group-231
Anthribinae_sp2	1	Group-2	Group-2	Group-82	Group-239
Anthribinae_sp3	1	Group-1	Group-1	Group-34	Group-214
*Eucorynus*_sp1	2	Group-4	Group-4	Group-21	Group-133
*Xenocerus*_sp1	2	Group-3	Group-3	Group-198	Group-107
*Xenocerus*_sp1	2	Group-5	Group-3	Group-199	Group-109
Apionini_sp1	17	Group-20	Group-19	Group-224	Group-137
Apionini_sp4	3	Group-20	Group-20	Group-225	Group-83
Apionini_sp5	2	Group-23	Group-23	Group-64	Group-193
Apionini_sp6	2	Group-19	Group-18	Group-74	Group-181
Apionini_sp7	2	Group-18	Group-17	Group-73	Group-165
*Baryrhynchus*_sp1	5	Group-21	Group-21	Group-80	Group-151
*Baryrhynchus*_sp2	2	Group-22	Group-22	Group-81	Group-234
Attelabinae_sp1	1	Group-12	Group-11	Group-69	Group-227
Attelabinae_sp2	1	Group-14	Group-13	Group-70	Group-35
Attelabinae_sp4	6	Group-10	Group-9	Group-30	Group-74
Attelabinae_sp5	1	Group-15	Group-14	Group-24	Group-54
*Paratrachelophorus*_sp1	5	Group-16	Group-15	Group-71	Group-124
Rhynchitinae_sp1	8	Group-8	Group-7	Group-182	Group-230
Rhynchitinae_sp3	1	Group-9	Group-8	Group-230	Group-158
Rhynchitinae_sp3	1	Group-9	Group-8	Group-231	Group-159
Rhynchitinae_sp4	8	Group-7	Group-9	Group-56	Group-176
Rhynchitinae_sp6	1	Group-11	Group-10	Group-58	Group-202
Rhynchitinae_sp7	2	Group-13	Group-12	Group-18	Group-97
Rhynchitinae_sp8	2	Group-17	Group-16	Group-57	Group-114
Aclees_cribratus	2	Group-112	Group-107	Group-213	Group-80
Aclees_cribratus	1	Group-112	Group-107	Group-212	Group-67
Baridini_sp1	1	Group-229	Group-220	Group-135	Group-212
*Calomycterus*_sp1	4	Group-228	Group-219	Group-250	Group-191
*Calomycterus*_sp1	2	Group-228	Group-219	Group-251	Group-191
Catapionus_mopsus	3	Group-27	Group-27	Group-94	Group-75
*Cionus*_sp1	4	Group-226	Group-217	Group-88	Group-229
*Cionus*_sp3	6	Group-225	Group-216	Group-87	Group-157
Cleonini_sp1	1	Group-224	Group-215	Group-43	Group-48
Conoderinae_sp1	3	Group-213	Group-204	Group-13	Group-180
Conoderinae_sp10	1	Group-223	Group-214	Group-66	Group-141
Conoderinae_sp11	1	Group-222	Group-213	Group-67	Group-127
Conoderinae_sp12	1	Group-221	Group-212	Group-134	Group-112
Conoderinae_sp13	1	Group-220	Group-211	Group-105	Group-93

Conoderinae_sp14	2	Group-219	Group-210	Group-195	Group-199
Conoderinae_sp15	2	Group-218	Group-209	Group-194	Group-185
Conoderinae_sp16	2	Group-217	Group-208	Group-110	Group-172
Conoderinae_sp17	1	Group-216	Group-207	Group-101	Group-155
Conoderinae_sp18	1	Group-215	Group-206	Group-65	Group-238
Conoderinae_sp19	6	Group-214	Group-205	Group-19	Group-228
Conoderinae_sp3	1	Group-212	Group-203	Group-25	Group-208
Conoderinae_sp4	1	Group-211	Group-202	Group-72	Group-233
Conoderinae_sp5	1	Group-210	Group-201	Group-100	Group-225
Conoderinae_sp6	3	Group-209	Group-200	Group-188	Group-26
Conoderinae_sp7	2	Group-208	Group-200	Group-187	Group-7
Conoderinae_sp8	1	Group-207	Group-199	Group-160	Group-70
Conoderinae_sp9	2	Group-206	Group-198	Group-151	Group-41
Cosmopolites_sordidus	1	Group-205	Group-197	Group-20	Group-220
Cotasterosoma_coronus	4	Group-49	Group-48	Group-214	Group-122
Cotasterosoma_coronus	3	Group-49	Group-48	Group-215	Group-168
*Crypturgus*_sp1	3	Group-204	Group-196	Group-12	Group-236
*Curculio*_sp1	2	Group-203	Group-195	Group-45	Group-217
Curculioninae_sp1	6	Group-202	Group-194	Group-53	Group-209
Cyphicerini_sp1	5	Group-201	Group-193	Group-146	Group-46
Cyphicerini_sp2	5	Group-200	Group-173	Group-221	Group-73
*Cyrtepistomus*_sp1	2	Group-199	Group-192	Group-38	Group-150
Dactylotinomorphus_sp1	3	Group-198	Group-191	Group-156	Group-3
*Dactylotus*_sp1	3	Group-197	Group-190	Group-155	Group-207
*Dactylotus*_sp2	1	Group-196	Group-189	Group-154	Group-216
Dermatoxenus_helleri	1	Group-194	Group-187	Group-137	Group-187
*Dermatoxenus*_helleri	1	Group-194	Group-188	Group-138	Group-198
Devernodes_alkippe	7	Group-39	Group-39	Group-196	Group-38
Devernodes_alkippe	5	Group-48	Group-40	Group-197	Group-129
*Dryocoetes*_sp1	4	Group-193	Group-186	Group-140	Group-64
Dryophthorinae_sp1	1	Group-192	Group-185	Group-19	Group-152
Dryophthorinae_sp2	3	Group-191	Group-184	Group-8	Group-169
Dryophthorini_sp1	3	Group-190	Group-183	Group-16	Group-205
Dryophthorini_sp3	3	Group-198	Group-182	Group-15	Group-179
Ectatorhinus_adamsii	3	Group-38	Group-38	Group-22	Group-131
Entiminae_sp1	1	Group-188	Group-181	Group-232	Group-118
Entiminae_sp1	1	Group-188	Group-181	Group-233	Group-118
Entiminae_sp10	3	Group-187	Group-180	Group-55	Group-197
Entiminae_sp11	6	Group-186	Group-179	Group-96	Group-184
Entiminae_sp12	1	Group-185	Group-178	Group-95	Group-110
Entiminae_sp13	1	Group-184	Group-177	Group-124	Group-86
Entiminae_sp14	2	Group-183	Group-176	Group-77	Group-138
Entiminae_sp15	2	Group-182	Group-175	Group-125	Group-123
Entiminae_sp16	2	Group-181	Group-174	Group-131	Group-30

Entiminae_sp17	3	Group-180	Group-173	Group-220	Group-12
Entiminae_sp18	3	Group-179	Group-173	Group-219	Group-71
Entiminae_sp19	1	Group-178	Group-172	Group-207	Group-49
Entiminae_sp2	6	Group-177	Group-171	Group-6	Group-145
Entiminae_sp20	1	Group-176	Group-170	Group-226	Group-163
Entiminae_sp20	1	Group-176	Group-170	Group-227	Group-164
Entiminae_sp21	2	Group-175	Group-169	Group-119	Group-206
Entiminae_sp22	3	Group-174	Group-168	Group-118	Group-186
Entiminae_sp23	6	Group-173	Group-167	Group-123	Group-113
Entiminae_sp25	6	Group-114	Group-110	Group-217	Group-143
Entiminae_sp3	2	Group-172	Group-166	Group-76	Group-99
Entiminae_sp4	2	Group-171	Group-165	Group-228	Group-115
Entiminae_sp4	2	Group-171	Group-165	Group-229	Group-116
Entiminae_sp5	1	Group-170	Group-164	Group-168	Group-189
Entiminae_sp6	1	Group-169	Group-163	Group-167	Group-204
Entiminae_sp7	1	Group-168	Group-162	Group-114	Group-162
Entiminae_sp8	1	Group-167	Group-161	Group-113	Group-178
Entiminae_sp9	1	Group-166	Group-160	Group-54	Group-232
Eugnathus_distinctus	3	Group-165	Group-159	Group-40	Group-192
Eugnathus_sp1	8	Group-164	Group-158	Group-248	Group-182
Eugnathus_sp1	5	Group-164	Group-158	Group-249	Group-182
Eusomidius_sp1	12	Group-163	Group-157	Group-238	Group-53
Eusomidius_sp1	1	Group-163	Group-157	Group-239	Group-53
Eusomidius_sp1	4	Group-163	Group-157	Group-240	Group-53
Eusomidius_sp1	5	Group-163	Group-157	Group-241	Group-53
Euwallacea_interjectus	5	Group-162	Group-156	Group-29	Group-111
Gasteroclisus_arcurostris	1	Group-161	Group-155	Group-42	Group-21
*Geotragus*_himalayanus	2	Group-103	Group-99	Group-33	Group-98
*Geotragus*_sp.CG311	1	Group-160	Group-154	Group-48	Group-103
*Hylobius*_sp1	3	Group-159	Group-153	Group-133	Group-45
*Hyperomias*_sp.1RL	2	Group-89	Group-87	Group-49	Group-6
*Hyperomias*_sp2	2	Group-93	Group-91	Group-169	Group-58
*Ips*_sp1	6	Group-158	Group-152	Group-5	Group-18
*Lagenolobus*_sp1	1	Group-157	Group-151	Group-97	Group-125
Leptomias_acutus	3	Group-65	Group-63	Group-242	Group-50
Leptomias_acutus	2	Group-65	Group-63	Group-243	Group-50
Leptomias_clarus	8	Group-63	Group-62	Group-157	Group-149
Leptomias_clavicrus	11	Group-98	Group-96	Group-202	Group-27
Leptomias_crinitarsus	11	Group-108	Group-103	Group-75	Group-63
Leptomias_depressus	2	Group-81	Group-79	Group-193	Group-2
Leptomias_erectus	4	Group-86	Group-84	Group-26	Group-78
Leptomias_foveicollis	35	Group-153	Group-148	Group-47	Group-91
Leptomias_hirsutus	12	Group-99	Group-82	Group-204	Group-44
Leptomias_huangi	118	Group-111	Group-106	Group-150	Group-42

Leptomias_kangmarensis	10	Group-62	Group-61	Group-174	Group-28
Leptomias_kangmarensis	13	Group-64	Group-61	Group-176	Group-135
Leptomias_kangmarensis	1	Group-156	Group-61	Group-175	Group-174
Leptomias_kingdonwardi	5	Group-67	Group-65	Group-192	Group-195
Leptomias_lineatus	11	Group-106	Group-101	Group-50	Group-166
Leptomias_mangkamensis	3	Group-97	Group-95	Group-61	Group-105
Leptomias_micans	32	Group-105	Group-100	Group-178	Group-69
Leptomias_midlineatus	4	Group-98	Group-96	Group-200	Group-25
Leptomias_midlineatus	4	Group-100	Group-96	Group-201	Group-61
Leptomias_sagaensis	12	Group-79	Group-77	Group-153	Group-36
Leptomias_semicircularis	78	Group-104	Group-63	Group-218	Group-9
Leptomias_siahus	5	Group-109	Group-104	Group-210	Group-136
Leptomias_siahus	2	Group-110	Group-105	Group-211	Group-47
*Leptomias*_sp._6_ZM-2022a	7	Group-83	Group-81	Group-51	Group-5
*Leptomias*_sp.12RL	3	Group-66	Group-64	Group-236	Group-14
*Leptomias*_sp.13RL	2	Group-88	Group-86	Group-149	Group-62
*Leptomias*_sp.15RL	2	Group-84	Group-82	Group-203	Group-147
*Leptomias*_sp.17RL	3	Group-66	Group-64	Group-237	Group-14
*Leptomias*_sp.18RL	2	Group-82	Group-80	Group-185	Group-8
*Leptomias*_sp.19RL	4	Group-80	Group-78	Group-158	Group-85
*Leptomias*_sp.25RL	2	Group-76	Group-74	Group-244	Group-23
*Leptomias*_sp.27RL	2	Group-75	Group-73	Group-180	Group-134
*Leptomias*_sp.28RL	3	Group-74	Group-72	Group-161	Group-144
*Leptomias*_sp.29RL	4	Group-73	Group-71	Group-162	Group-24
*Leptomias*_sp.2RL	4	Group-95	Group-93	Group-128	Group-128
*Leptomias*_sp.30RL	3	Group-72	Group-70	Group-159	Group-76
*Leptomias*_sp.3RL	2	Group-96	Group-94	Group-172	Group-96
*Leptomias*_sp.9RL	2	Group-76	Group-74	Group-245	Group-55
*Leptomias*_sp.9RL	13	Group-76	Group-74	Group-246	Group-23
*Leptomias*_sp.9RL	3	Group-76	Group-74	Group-247	Group-23
*Leptomias*_sp32RL	1	Group-155	Group-150	Group-52	Group-153
*Leptomias*_sp33RL	1	Group-154	Group-149	Group-186	Group-171
*Leptomias*_sp5RL	6	Group-64	Group-61	Group-235	Group-82
Leptomias_tsanghoensis	8	Group-71	Group-69	Group-129	Group-170
Leptomias_undulans	2	Group-101	Group-97	Group-181	Group-19
Leptomias_viridicantis	20	Group-107	Group-102	Group-179	Group-142
Lixini_sp1	1	Group-152	Group-147	Group-41	Group-95
*Lixus*_sp1	1	Group-151	Group-146	Group-166	Group-196
*Lixus*_sp2	1	Group-150	Group-145	Group-165	Group-183
*Lixus*_sp3	1	Group-149	Group-144	Group-44	Group-167
*Lobotrachelus*_sp1	4	Group-148	Group-143	Group-31	Group-173
Mecopini_sp1	12	Group-147	Group-142	Group-35	Group-10
Merus_flavosignatus	1	Group-146	Group-141	Group-90	Group-31
*Merus*_sp1	3	Group-145	Group-140	Group-89	Group-66

Microplinthus_emeishanicus	1	Group-227	Group-218	Group-79	Group-17
Molytinae_sp1	1	Group-144	Group-139	Group-132	Group-201
Molytinae_sp2	1	Group-143	Group-138	Group-115	Group-161
Molytinae_sp3	1	Group-142	Group-137	Group-116	Group-175
Molytinae_sp4	1	Group-141	Group-136	Group-4	Group-226
Molytinae_sp5	1	Group-140	Group-135	Group-63	Group-235
Molytinae_sp6	1	Group-139	Group-134	Group-92	Group-210
Molytinae_sp7	1	Group-138	Group-133	Group-62	Group-219
Molytinae_sp8	1	Group-137	Group-132	Group-7	Group-56
*Moreobaris*_sp1	1	Group-136	Group-131	Group-223	Group-194
*Moreobaris*_sp2	10	Group-136	Group-131	Group-222	Group-84
Morimotodes_ismene	7	Group-26	Group-26	Group-84	Group-79
Morimotodes_ismene	6	Group-33	Group-33	Group-85	Group-200
Morimotodes_ismene	9	Group-37	Group-37	Group-86	Group-100
Morimotodes_ismene	2	Group-40	Group-41	Group-141	Group-22
Morimotodes_ismene	9	Group-43	Group-44	Group-183	Group-37
Morimotodes_ismene	1	Group-51	Group-50	Group-184	Group-198
Myllocerus_viridis	4	Group-135	Group-130	Group-46	Group-88
Nedyus_quadrimaculatus	1	Group-134	Group-129	Group-109	Group-32
*Niphades*_sp1	2	Group-133	Group-128	Group-107	Group-126
*Niphades*_sp2	2	Group-132	Group-127	Group-106	Group-140
Niphades_variegatus	12	Group-131	Group-126	Group-108	Group-102
Niphadomimus_alcyone	1	Group-36	Group-36	Group-205	Group-215
Niphadomimus_alcyone	1	Group-46	Group-36	Group-206	Group-52
Niphadomimus_calaeno	1	Group-45	Group-46	Group-191	Group-59
Niphadomimus_sterope	2	Group-34	Group-34	Group-173	Group-106
Notaris_kozlovi	15	Group-24	Group-24	Group-170	Group-39
Notaris_kozlovi	7	Group-25	Group-25	Group-171	Group-177
Notaris_kozlovi	1	Group-29	Group-29	Group-1	Group-132
Notaris_kozlovi	1	Group-30	Group-30	Group-2	Group-187
Notaris_kozlovi	8	Group-44	Group-45	Group-144	Group-20
Notaris_kozlovi	1	Group-47	Group-47	Group-3	Group-11
Notaris_kozlovi	1	Group-50	Group-49	Group-171	Group-60
Odoiporus_longicollis	15	Group-130	Group-125	Group-60	Group-119
Odontomias_crassus	2	Group-68	Group-66	Group-142	Group-92
Odontomias_nigrolatus	5	Group-69	Group-67	Group-143	Group-57
*Odontomias*_sp.1RL	3	Group-94	Group-92	Group-127	Group-90
*Odontomias*_sp.2RL	4	Group-91	Group-89	Group-126	Group-146
*Odontomias*_sp.3RL	5	Group-90	Group-88	Group-117	Group-34
*Odontomias*_sp.4RL	3	Group-78	Group-76	Group-147	Group-81
*Odontomias*_sp5	2	Group-92	Group-90	Group-152	Group-16
Pachynotus_lampoglobus	2	Group-70	Group-68	Group-32	Group-120
Pachynotus_pilosus	2	Group-102	Group-98	Group-17	Group-156
Peribleptus_foveostriatus	1	Group-129	Group-124	Group-136	Group-15

Peribleptus_scalptus	3	Group-127	Group-122	Group-189	Group-77
Peribleptus_scalptus	4	Group-128	Group-123	Group-190	Group-68
Phytoscaphus_chloroticus	3	Group-126	Group-121	Group-164	Group-190
Phytoscaphus_ciliatus	3	Group-125	Group-120	Group-121	Group-154
Phytoscaphus_decorus	2	Group-124	Group-119	Group-122	Group-160
Phytoscaphus_kaulbacki	9	Group-123	Group-116	Group-209	Group-237
Phytoscaphus_lineatus	15	Group-122	Group-118	Group-120	Group-1
*Phytoscaphus*_sp2	2	Group-121	Group-117	Group-68	Group-240
*Phytoscaphus*_sp3	2	Group-120	Group-116	Group-208	Group-213
*Phytoscaphus*_sp4	2	Group-119	Group-115	Group-163	Group-221
*Phytoscaphus*_sp5	5	Group-118	Group-114	Group-78	Group-188
*Platymycteropsis*_sp1	16	Group-117	Group-113	Group-39	Group-211
Ptochus_potanini	6	Group-116	Group-112	Group-130	Group-222
*Ptochus*_sp1	1	Group-115	Group-111	Group-91	Group-117
*Ptochus*_sp2	3	Group-114	Group-109	Group-216	Group-130
Rhynchophorini_sp1	5	Group-113	Group-108	Group-59	Group-139
Scolytinae_sp1	1	Group-61	Group-60	Group-37	Group-13
Scolytinae_sp2	1	Group-60	Group-59	Group-28	Group-72
Scolytinae_sp3	2	Group-59	Group-58	Group-36	Group-51
Scolytinae_sp4	2	Group-58	Group-57	Group-139	Group-218
Sitona_cylindricollis	9	Group-57	Group-56	Group-11	Group-223
*Sitona*_sp2	7	Group-56	Group-55	Group-10	Group-94
Sitophilus_oryzae	1	Group-55	Group-54	Group-104	Group-121
Sitophilus_zeamais	8	Group-42	Group-43	Group-103	Group-4
Trachyphloeoides_io	5	Group-32	Group-32	Group-93	Group-29
*Triangulomias*_sp.1RL	3	Group-77	Group-75	Group-177	Group-104
*Triangulomias*_sp.2RL	2	Group-77	Group-75	Group-177	Group-104
*Triangulomias*_sp.3RL	3	Group-85	Group-83	Group-148	Group-203
*Triangulomias*_sp.4RL	2	Group-77	Group-75	Group-177	Group-104
Trichalophus_caudiculatus	3	Group-31	Group-31	Group-98	Group-65
Trichalophus_scylla	3	Group-35	Group-35	Group-99	Group-33
*Trichalophus*_tibetanus	4	Group-28	Group-28	Group-111	Group-43
*Trichalophus*_tibetanus	3	Group-41	Group-28	Group-112	Group-101
*Trochorhopalus*_sp1	5	Group-53	Group-52	Group-9	Group-10
Tychius_oriens	2	Group-52	Group-51	Group-23	Group-108
*Xizanomias*_sp.1RL	2	Group-87	Group-85	Group-102	Group-148
Xylosandrus_crassiusculus	4	Group-54	Group-53	Group-27	Group-24

Section S1. Checklist of Weevil Species from the Qinghai–Xizang Plateau

Anthribidae Billberg, 1820
Anthribinae Billberg, 1820
Tribe Ecelonerini Lacordaire, 1865
genus *Disphaerona* Jordan, 1902
chinensis Frieser, 1995

Tribe Platystomini Pierce, 1916
genus *Phloeobius* Schoenherr, 1823
lepticornis Jordan, 1912

Tribe Tropiderini Lacordaire, 1865
genus *Merarius* Fairmaire, 1889
davidis Fairmaire, 1889
genus *Sphinctotropis* H.J. Kolbe, 1895
scabrosa Frieser, 1983

Tribe Xylinadini Lacordaire, 1865
genus *Xylinada* Berthold, 1827
annulipes (Jordan, 1895)

Choraginae Kirby, 1819
Tribe Araecerini Lacordaire, 1865
genus *Araecerus* Schoenherr, 1823
fasciculatus (DeGeer, 1775)

Attelabidae Billberg, 1820
Attelabinae Billberg, 1820
Tribe Apoderini Jekel, 1860
genus *Anisonychus* Voss, 1927
rugosus (Voss, 1927)
genus *Compsapoderus* Voss, 1927
minimus (Roelofs, 1874)
lepidulus (Voss, 1927)
seclusus (Faust, 1898)
genus *Mordkovitshirhinus* Legalov, 2003
yunnanicus Legalov, 2003
genus *Apoderus* Olivier, 1807
fidus Faust, 1890
pseudofidus Legalov, 2003
rugicollis Schilsky, 1906
volkovitshi Legalov, 2003

genus *Centrocorynus* Jekel, 1860

scutellaris (Gyllenhal, 1833)

genus *Heterapoderus* Voss, 1927

ageniculatus Legalov, 2007

geniculatus (Jekel, 1860)

genus *Leptapoderus* Jekel, 1860

vossi (Biondi, 2001)

yunlingensis Legalov, 2007

proprius Legalov, 2007

affinis (Schilsky, 1906)

balteatus (Roelofs, 1874)

dubatolovi Legalov, 2003

inbalteatus Legalov, 2007

friedrichi Voss, 1956

genus *Paracycnotrachelus* Voss, 1924

breviusculus Voss, 1929

chinensis (Jekel, 1860)

genus Cycnotrachelodes Voss, 1955

sitchuanensis Legalov, 2003

cyanopterus (Motschulsky, 1860)

genus *Paratrachelophorus* Voss, 1924

brachmanus Voss, 1924

daliensis Legalov, 2003

potanini (Faust, 1890)

gigas Legalov, 2003

marsi Legalov, 2003

nodicornis Voss, 1924

genus *Physapoderus* Jekel, 1860

crucifer (Heller, 1922)

genus *Maculphrysus* Legalov, 2003

inspersus (Voss, 1929)

yunnanicus Legalov, 2003

genus *Pseudophrysus* Legalov, 2003

parvulus (Voss, 1928)

genus *Agomadaranus* Voss, 1958

bistrispinosus (Faust, 1894)

coniceps (Voss, 1926)

melanostictoides (Legalov, 2003)

kryzhanovskyi (Legalov, 2003)

genus *Paroplapoderus* Voss, 1926

punctatus Pic, 1928

tentator tentator (Faust, 1894)

genus *Phymatapoderus* Voss, 1926

euflavimanus Legalov, 2003

sitchuanensis Legalov, 2003

genus *Tomapoderus* Voss, 1926

pici (Legalov, 2007)

cyclops (Faust, 1894)

Tribe Attelabini Billberg, 1820

genus *Attelabus* Linnaeus, 1758

sichuanensis Legalov & N. Liu, 2005

genus *Cyrtolabus* Voss, 1925

amitinus (Voss, 1932)

nanpingensis Legalov, 2007

Tribe Euopini Voss, 1925

genus *Euops* Schoenherr, 1839

nielamus (Legalov & N. Liu, 2005)

yunnanensis Liang, 2005

verrucosus (Legalov & X. Zhang, 2007)

asiaticus asiaticus (Legalov & N. Liu, 2005)

carinatus (Legalov & X. Zhang, 2007)

chinensis Voss, 1922

yunnanicus (Legalov, 2003)

pseudopolitus (Legalov, 2003)

moanus (Legalov, 2003)

lushuensis (Legalov & N. Liu, 2005)

pyralis (Legalov & N. Liu, 2005)

tibetanus (Legalov & N. Liu, 2005)

viridis (Legalov, 2003)

fugongensis J. Wang & Liang, 2008

jiuzhaiensis Liang, 2005

rasuwanus (Legalov, 2003)

sichuanensis (Legalov & N. Liu, 2005)

Tribe Euscelophilini Voss, 1925

genus *Euscelophilus* Voss, 1925

fasciatus (X.-C. Zhang, 1993)

burmanus G.A.K. Marshall, 1948

chinensis (Schilsky, 1906)

denticulatus X.-C. Zhang, 1995

gibbicollis (Schilsky, 1906)

gaoligongensis Xie & Liang, 2008

camelus Voss, 1937

rugulosus X.-C. Zhang, 1995

genus *Trachelolabus* Jekel, 1860

floridus (X.-C. Zhang, 1993)

longispinus (X.-C. Zhang, 1993)
whitei Jekel, 1860

Tribe Lamprolabini Voss, 1925
genus *Henicolabus* Voss, 1925
hypomelas Fairmaire, 1878
genus *Humerilabus* Legalov, 2003
fausti (Voss, 1925)
genus *Isolabus* Voss, 1925
intrusus Voss, 1956
magnus Voss, 1925
genus *Lamprolabus* Jekel, 1860
tibetanus Legalov & N. Liu, 2005

Tribe Paramecolabini Legalov, 2003
genus *Paramecolabus* Jekel, 1860
castaneicolor (Jekel, 1860)

Rhynchitinae Gistel, 1848
Tribe Auletini Desbrochers des Loges, 1908
genus *Auletobius* Desbrochers des Loges, 1869
habashanensis Legalov, 2007
baishuiensis Legalov, 2010
genus *Pseudomesauletes* Legalov, 2001
dundai Legalov, 2007
nanpingensis Legalov, 2007

Tribe Byctiscini Voss, 1923
genus *Aspidobyctiscus* Schilsky, 1903
nanpingensis Legalov, 2009
lacunipennis (Jekel, 1860)
niger Legalov, 2003
genus *Byctiscus* C.G. Thomson, 1859
bilineatoides Legalov, 2007
davidiani Legalov, 2003
kresli Legalov, 2003
impressus impressus (Fairmaire, 1900)
populi (Linnaeus, 1758)
princeps (Solsky, 1872)
thibetanus (Legalov, 2007)
rugosus (Gebler, 1829)
macros Legalov, 2004

Tribe Deporaini Voss, 1929

genus *Caenorhinus* C.G. Thomson, 1859
bicoloripes Legalov, 2007
dundai Legalov, 2007
atrorufus (Voss, 1942)
rusticapitus Legalov, 2007
marginellus (Faust, 1898)
genus *Deporaus* Samouelle, 1819
brunneoclavus Legalov, 2007
flaviclavus Legalov, 2007
gunlaishanensis Legalov, 2007
kangdingensis Legalov, 2007
mugezoensis Legalov, 2007
ruber Legalov & N. Liu, 2005

Tribe Eugnamptini Voss, 1930
genus *Neoeugnamptus* Legalov, 2003
habashanensis Legalov, 2007
cyaneus Legalov, 2007
julonxueshanicus (Legalov, 2007)
genus *Aderorhinus* Sharp, 1889
pedicellaris Voss, 1930
genus *Eugnamptobius* Voss, 1922
tessellatus (Voss, 1920)
genus *Eugnamptus* Schoenherr, 1839
kubani Legalov, 2007
sitshuanensis Legalov, 2003

Tribe Rhynchitini Gistel, 1848
genus *Auletomorphus* Voss, 1923
habashanensis Legalov, 2011
montanus Legalov, 2003
tonkinensis (Voss, 1924)
genus *Cneminvolvulus* Voss, 1960
schusteri (Formánek, 1912)
lepidus (Voss, 1930)
genus *Cyllorhynchites* Voss, 1930
indicus Legalov, 2003
cumulatus (Voss, 1930)
genus *Involvulus* Schrank, 1798
yanmensis Legalov, 2009
cupreus (Linnaeus, 1758)
gemma (Semenov & Ter-Minasian, 1937)
hauseri (Wagner, 1907)
dundai (Legalov, 2007)

kangdingensis (Legalov, 2007)
nigrocyaneus (Legalov, 2007)
genus *Mecorhis* Billberg, 1820
insolita Voss, 1924
kundensis Legalov, 2007
genus *Rubrrhynchites* Legalov, 2007
curvirostris Legalov, 2007
genus *Rhynchites* D.H. Schneider, 1791
fulgidus Faldermann, 1835
heros Roelofs, 1874
genus *Pustulorhinus* Legalov, 2003
yunnanicus Legalov, 2003

Brentidae Billberg, 1820
Apioninae Schoenherr, 1823
Tribe Apionini Schoenherr, 1823
genus *Protoceratapion* Wanat, 1995
tibetanum (Balfour-Browne, 1944)
genus *Pseudapion* Schilsky, 1906
fulvirostre (Gyllenhal, 1833)
genus *Cyanapion* Bokor, 1923
gyllenhalii (Kirby, 1808)
genus *Eutrichapion* Reitter, 1916
hingstoni (Balfour-Browne, 1944)
viciae (Paykull, 1800)
genus *Hemitrichapion* Voss, 1959
tschernovi tschernovi (Ter-Minasian, 1973)
genus *Synapion* Schilsky, 1902
kaulbacki (Balfour-Browne, 1944)
kozlovi Korotyaev, 1988
genus *Pseudoprotapion* Ehret, 1990
paracoeleste (Balfour-Browne, 1944)
genus *Pseudopiezotrachelus* Wagner, 1907
collaris Schilsky, 1906

Brentinae Billberg, 1820
Tribe Brentini Billberg, 1820
genus *Baryrhynchus* Lacordaire, 1865
miles (Boheman, 1845)
cratus R. Zhang, 1993
dehiscens (Gyllenhal, 1833)
planus R. Zhang, 1993
genus *Agriorrhynchus* Power, 1878
quadrituberculatus Senna, 1892

Curculionidae Latreille, 1802 s.l.

Brachycerinae Billberg, 1820

Tribe Erirhinini Schoenherr, 1825

genus *Notaris* Germar, 1817

distans Faust, 1890

genus *Notaroides* Korotyaev, 1979

brevirostris Korotyaev, 1979

genus *Echinocnemus* Schoenherr, 1843

dorsalis Chevrolat, 1879

Conoderinae Schoenherr, 1833

Tribe Baridini Schoenherr, 1836

genus *Pharcidobaris* Morimoto & Yoshihara, 1996

miyamotoi Morimoto & Yoshihara, 1996

pilosa (Roelofs, 1875)

genus *Psilarthrus* G.A.K. Marshall, 1948

coracinus G.A.K. Marshall, 1948

genus *Cosmobaris* Casey, 1920

scolopacea (Germar, 1819)

genus *Moreobaris* Morimoto & Yoshihara, 1996

deplanata (Roelofs, 1875)

Tribe Amalini Wagner, 1936

genus *Nedyus* Schoenherr, 1825

quadrimaculatus (Linnaeus, 1758)

genus *Coeliodes* Schoenherr, 1837

tibetanus Yoshitake & Huang

Tribe Cnemogonini Colonnelli, 1979

genus *Coeliosomus* Motschulsky, 1858

ustulatus (Voss, 1958)

genus *Mecysmoderes* Schoenherr, 1837

yunnanensis (J. Huang, R. Zhang & Pelsue)

fulvus Roelofs, 1875

genus *Neophytobius* Wagner, 1936

quadrinodosus (Gyllenhal, 1813)

Tribe Scleropterini Schultze, 1902

genus *Scleropterus* Schoenherr, 1825

antoni Korotyaev, 2008

genus *Tibetiellus* Korotyaev, 1980

winnipooh Korotyaev, 1980

genus *Rutidosoma* Stephens, 1831

alexanderi Korotyaev, 2008

Tribe Cnemogonini Colonnelli, 1979
genus *Xenysmoderes* Colonnelli, 1992
armirufus G.A.K. Marshall, 1948

Cossoninae Schoenherr, 1825
Tribe Cossonini Schoenherr, 1825
genus *Stereoborus* Wollaston, 1873
chinensis Omar & R. Zhang, 2007

Tribe Onycholipini Wollaston, 1873
genus *Stenoscelodes* Konishi, 1962
tibetanus R. Zhang & G. Osella, 1992
genus *Hexarthrum* Wollaston, 1860
chaoi R. Zhang & G. Osella, 1995
genus *Stenoscelis* Wollaston, 1861
recava R. Zhang, 1995

Tribe Rhyncolini Gistel, 1848
genus *Xenomimetes* Wollaston, 1873
alni Konishi, 1955
destructor Wollaston, 1873

Curculioninae Latreille, 1802
Tribe Anthonomini C. G. Thomson, 1859
genus *Anthonomus* Germar, 1817
costipennis Fairmaire, 1889
rubi (Herbst, 1795)
genus *Coccotorus* LeConte, 1876
beijingensis Lin & Li, 1990

Tribe Cionini Schoenherr, 1825
genus *Nanomicrophyes* Pic, 1908
belousovi Davidian & Korotyaev, 2017
kabaki Davidian & Korotyaev, 2017
oblongus Davidian & Korotyaev, 2017
genus *Cionus* Clairville, 1798
yunnanensis Košťál & Caldara, 2019

Tribe Curculionini Latreille, 1802
genus *Curculio* Linnaeus, 1758
aino (Kôno, 1930)
pylzovi (Smirnov, 1913)

nigritudinis Pelsue & Caldara, 2013
bullabrevis Pelsue & R. Zhang, 2003
careoparvus Pelsue & R. Zhang, 2003
dieckmanni (Faust, 1887)
hobbsi Pelsue & R. Zhang, 2003
megadens Pelsue & R. Zhang, 2003
misellus (Heller, 1927)
tarae Pelsue & R. Zhang, 2002
genus Ergania Pascoe, 1882
doriae yunnana (Heller, 1927)
genus Pseudoculio Pelsue & O'Brien, 2011
crinitus Pelsue & O'Brien, 2011

Tribe Rhamphini Rafinesque, 1815
genus *Rhamphus* Clairville, 1798
tsaidamicus Korotyaev, 1984

Tribe Tychiini C. G. Thomson, 1859
genus *Demimaea* Pascoe, 1870
vau Voss, 1953
genus *Tychius* Germar, 1817
oriens A. Hoffmann, 1964
thompsoni Caldara, 1990
vossi Caldara, 1990

Dryophthorinae Schoenherr, 1825
Tribe Orthognathini Lacordaire, 1865
genus *Sipalinus* G.A.K. Marshall, 1943
gigas gigas (Fabricius, 1775)
yunnanensis Vaurie, 1971

Tribe Rhynchophorini Schoenherr, 1833
genus *Sitophilus* Schoenherr, 1838
oryzae (Linnaeus, 1763)
zeamais Motschulsky, 1855
genus *Rhynchophorus* Herbst, 1795
ferrugineus (Olivier, 1791)
genus *Odoiporus* Chevrolat, 1885
longicollis (Olivier, 1807)

Entiminae Schoenherr, 1823
Tribe Blosyrini Lacordaire, 1863
genus *Dactylotus* Schoenherr, 1847
bistriolatus R. Zhang, 1993

concavus R. Zhang, 1993
expansus R. Zhang, 1993
exsertus R. Zhang, 1993
lubricus R. Zhang, 1993
polytrichus R. Zhang, 1993
ruidus R. Zhang, 1993
scrobiculus R. Zhang, 1993
sedakoffi Schoenherr, 1847
semipubens Faust, 1887
trisulcus R. Zhang, 1993
ventralis R. Zhang, 1993
curvativus R. Zhang
dorsalis R. Zhang, 1996
rugolosus R. Zhang, 1996
latiusculus R. Zhang, 1996
genus *Kamius* Korotyaev, 1996
kozlovi Korotyaev, 1979
genus *Dactylotinomorphus* Davidian, 2021
egenus (Faust, 1887)
gonghensis (R. Zhang, 1993)
ligulatus (R. Zhang, 1993)
nitidulus (Faust, 1895)
roborovskyi (Faust, 1887)
xinghaiensis (R. Zhang, 1993)
genus *Blosyrodes* Jekel, 1875
pubescens G.A.K. Marshall, 1916

Tribe Byrsopagini Lacordaire, 1863
genus *Acanthalophus* Morimoto, 2015
bicaudatus Colonnelli, 2018
genus *Pseudalophus* Suvorov, 1915
asyae Davidian & Arzanov, 2019
angulatus Davidian & Arzanov, 2020
bajtenovi Davidian & Arzanov, 2019
ballioni ballioni Davidian & Arzanov, 2019
ballioni guttatus Davidian & Arzanov, 2019
belousovi Davidian & Arzanov, 2019
boleslavi Davidian & Arzanov, 2019
borisi Davidian & Arzanov, 2019
brevirostris Davidian & Arzanov, 2020
fausti Davidian & Arzanov, 2019
furcatus Davidian & Arzanov, 2019
frontalis Davidian & Arzanov, 2019
kabaki Davidian & Arzanov, 2019

kandingianus Davidian & Arzanov, 2019
korolevi Davidian & Arzanov, 2020
kozlovi Davidian & Arzanov, 2019
marginatus (Faust, 1887)
muliensis meridionalis Davidian & Arzanov, 2020
paratibetanus Davidian & Arzanov, 2019
przewalskii Davidian & Arzanov, 2019
reitteri Davidian & Arzanov, 2019
rudigeri Davidian & Arzanov, 2019
rufimanus Davidian & Arzanov, 2019
scylla (Grebennikov, 2015)
scylloides Davidian & Arzanov, 2020
suvorovi Davidian & Arzanov, 2019
tibetanus Suvorov, 1915
tuberculatus Davidian & Arzanov, 2019
genus *Trichalophus* LeConte, 1876
pacatus (Faust, 1890)

Tribe Cneorhinini Lacordaire, 1863
genus *Callirhopalus* Hochhuth, 1851
sedakowii Hochhuth, 1851
genus *Catapionus* Schoenherr, 1842
mopsus Grebennikov, 2016
tibetanus Suvorov, 1915
genus *Dermatoxenus* G.A.K. Marshall, 1916
caesicollis (Gyllenhal, 1833)
helleri G.A.K. Marshall, 1916
sexnodosus Voss, 1932

Tribe Cyphicerini Lacordaire, 1863
genus *Heteroptochus* Faust, 1886
temperans Faust, 1890
genus *Lagenolobus* Faust, 1887
lineolatus Hustache, 1928
tibetanus (Formánek, 1916)
genus *Corigetus* Desbrochers des Loges, 1872
marmoratus Desbrochers des Loges, 1873
分布：青海
genus *Neomyllocerus* Voss, 1934
hedini (G.A.K. Marshall, 1934)
genus *Phrixopogon* G.A.K. Marshall, 1941
excisangulus (Reitter, 1900)
分布：西藏
genus *Ptochus* Schoenherr, 1826

indemnis Faust, 1890
koltzei Reitter, 1906
obliquesignatus Reitter, 1906
potanini Reitter, 1906
genus *Chloebius* Schoenherr, 1826
aksuanus Reitter, 1915
immeritus (Schoenherr, 1826)
genus *Phytoscaphus* Schoenherr, 1826
ciliatus Roelofs, 1873
chloroticus Boheman, 1843
decorus Pajni, 1990
lineatus Faust, 1891
Kaulbacki Pajni, 1990

Tribe Episomini Lacordaire, 1863
genus *Episomus* Schoenherr, 1823
appendiculatus Faust, 1897

Tribe Ophryastini Lacordaire, 1863
genus *Deracanthus* Schoenherr, 1823
jakovlevi jakovlevi Suvorov, 1908
kukunorensis Suvorov, 1910
potanini Faust, 1890
roborowskii Suvorov, 1910

Tribe Otiorhynchini Schoenherr, 1826
genus *Otiorhynchus* Germar, 1822
russicus Stierlin, 1883

Tribe Polydrusini Schoenherr, 1823
genus *Polydrusus* Germar, 1817
analis Schilsky, 1910
longiceps Schilsky, 1912

Tribe Sitonini Gistel, 1848
genus *Sitona* Germar, 1817
amurensis Faust, 1882
cylindricollis cylindricollis Fåhraeus, 1840
hispidulus (Fabricius, 1777)
lineellus lineellus (Bonsdorff, 1785)
onerosus Faust, 1890
tessellatus Korotyaev, 1979
tibetanus Korotyaev, 1979
genus *Eugnathus* Schoenherr, 1834

distinctus Roelofs, 1873

Tribe Tanymecini Lacordaire, 1863

genus *Geotragus* Schoenherr, 1845

granulatus Chao, 1980

himalayanus Boheman, 1845

tuberculatus Y-Q. Chen, 1990

brevidens Ren, Alonso-Zarazaga & R. Zhang, 2013

rugosus Ren, Alonso-Zarazaga & R. Zhang, 2013

genus *Hyperomias* G.A.K. Marshall, 1916

adustus Y-Q. Chen, 1980

babaulti (Hustache, 1928)

bruneolineatus Zhang, 1996

convexus Zhang, 1996

curvatus Zhang, 1996

densus Y-Q. Chen, 1980

erectosetosus Y-Q. Chen, 1992

flavus Y-Q. Chen, 1980

foveatus Chao, 1980

fraxinus Y-Q. Chen, 1980

gracillis Y-Q. Chen, 1980

guinanensis Y-Q. Chen, 1980

immarginatus Y-Q. Chen, 1980

inordinatus Aslam, 1966

jiggyobensis Ren, Alonso-Zarazaga & Zhang, 2007

lacteolus Y-Q. Chen, 1980

lineolus Y-Q. Chen, 1980

marginatus Aslam, 1966

menyuanensis Y-Q. Chen, 1980

morulineolus Y.-Q. Chen, 1980

obscurus Y.-Q. Chen, 1980

rongbukensis Aslam, 1966

rotundicollis Chao, 1980

squamoopacus Y-Q. Chen, 1980

subdensus Y-Q. Chen, 1980

hengduanensis Y-Q. Chen, 1992

validus Chen, Y-Q. 1980

yushuensis Y-Q. Chen, 1980

genus *Lepropus* Schoenherr, 1823

chinensis (Fairmaire, 1889)

flavovittatus (Pascoe, 1881)

genus *Leptomias* Faust, 1886

acuminatus Aslam, 1961

acutus acutus Aslam, 1961

acutus zayuensis Chao, 1981
aeneus G.A.K. Marshall, 1955
alternans Chao, 1980
aphelocnemius Aslam, 1961
bispiculatus Y-Q. Chen, 1987
bicaudatus Y-Q. Chen, 1987
brevicornutus Chao, 1981
chagyabensis Chao & Y-Q. Chen, 1981
chaoi Y-Q. Chen, 1983
clavellatus Y-Q. Chen, 1992
interruptus (Aslam, 1969)
clarus Chao, 1980
crinitarsus Aslam, 1961
damxungensis Y-Q. Chen, 1981
elongatoides Y-Q. Chen, 1987
elongitus Chao, 1981
erectus Chao, 1981
pinguis (Aslam, 1969)
foveicollis (Voss, 1935)
foveolatus Chao, 1981
globicollis Aslam, 1961
globosus Y-Q. Chen, 1987
hirsutus Chao, 1981
huangi Chao, 1981
kangmarensis Chao, 1981
kingdonwardi G.A.K. Marshall, 1955
laticnemius Aslam, 1961
lhazeensis Y-Q. Chen, 1991
lineatus Aslam, 1961
longicollis Chao, 1981
longisetosus Chao, 1981
mainlingensis Chao, 1981
mangkamensis Chao & Y-Q. Chen, 1981
micans Chao, 1981: 544
midlineatus Chao, 1981
migo Alonso-Zarazaga & Ren, 2013
moxiensis Y-Q. Chen, 1992
nagarzeensis Y-Q. Chen, 1991
niger Y-Q. Chen, 1983
nigronitidus Y-Q. Chen, 1992
nubilus Y-Q. Chen, 1983
obconicus Chao, 1981
ochrolineatus Y-Q. Chen, 1987
opacus Chao, 1981

pandus Y-Q. Chen, 1981
pinnatus Y-Q. Chen, 1981
planocollis Chao, 1981
qamdoensis Chao & Y-Q. Chen, 1981
qomolangmaensis Y-Q. Chen, 1981
ramosus Y-Q. Chen, 1981
rubiginosus Y-Q. Chen, 1983
sagaensis Y-Q. Chen, 1981
schoenherri (Faust, 1881)
semicircularis Chao, 1981
shanlong Alonso-Zarazaga & Ren, 2013
siahus (Aslam, 1969)
squamosetosus Y-Q. Chen, 1981
strictus Y-Q. Chen, 1981
subaeneus Y-Q. Chen, 1981
sublongicollis Y-Q. Chen, 1987
submarginatus Chao, 1980
submidlineatus Y-Q. Chen, 1988
subundulans Y-Q. Chen, 1988
sulcus Y-Q. Chen, 1992: 842
tenuis Y-Q. Chen, 1992: 840
thibetanus (Faust, 1888)
triangulus Chao, 1981
trilineatus Chao, 1980
tsanghoensis Aslam, 1961
tuberculatus Chao & Y-Q. Chen, 1980
undulans G.A.K. Marshall, 1955
uniseries Y-Q. Chen, 1983
varians Y-Q. Chen, 1987
verticalis Ren, Zhang & Song, 2017
viridicantis Y-Q. Chen, 1988
viridilinearis Y-Q. Chen, 1984
zheduoshanensis Y-Q. Chen, 1992
yuhuensis Y-Q. Chen, 1992
yulongshanensis Y-Q. Chen, 1992
wenchuanensis Y-Q. Chen, 1992
clavicrus G.A.K. Marshall, 1955
genus *Meteutinopus* Zumpt, 1931
mongolicus (Faust, 1881)
opalescens (Faust, 1887)
genus *Odontomias* Y-Q. Chen, 1991
crassus (Y-Q. Chen, 1981)
dentatus (Y-Q. Chen, 1981)
latus (Chao, 1981)

minutus Y-Q. Chen, 1991
nigrolatus (Chao & Y-Q. Chen, 1981)
odontocnemus (Chao, 1981)
orbiculatus (Y-Q. Chen, 1983)
parvilatus (Y-Q. Chen, 1983)
subparvilatus Y-Q. Chen, 1991
genus *Pachynotus* Kollar & L. Redtenbacher, 1844
lampoglobus Chao & Y-Q. Chen, 1980
pilosus J-L. Ren, Ren & Zhang, 2024
arcuatus J-L. Ren, Ren & Zhang, 2024
genus *Piazomias* Schoenherr, 1840
desgodinsi J. Frivaldszky, 1892
kamicus Suvorov, 1915
kozlovi Suvorov, 1915
tibetanus Suvorov, 1915
genus *Triangulomias* Y-Q. Chen, 1991
acutiangulus Y-Q. Chen, 1991
angularus Y-Q. Chen, 1991
dicaris (Chao, 1980)
foveocollis B. Chen & Lan, 1997
impar (Chao & Y-Q. Chen, 1980)
linopennis B. Chen & Lan, 1997
microdentatus (Chao & Y-Q. Chen, 1981)
rotundus Y-Q. Chen, 1991
trianguloplatus (Chao, 1980)
waltoni (G.A.K. Marshall, 1916)
genus *Xizanomias* Chao, 1980
acutiangulus Chao, 1980
altus Chao, 1980
hohxilensis R. Zhang, 1996
latifrons Chao, 1980
magnus Chao, 1980
nyalamensis Chao, 1980
分布：西藏
genus *Xylinophorus* Faust, 1885
laetus (Faust, 1887)
stoeckleini Voss, 1943
tonsus (Faust, 1887)
prodromus Faust, 1885
genus *Lechrioderus* Faust, 1890
imbellus Faust, 1890
genus *Tainophthalmus* Desbrochers des Loges, 1873
subcarinatus Faust, 1883
genus *Chlorophanus* C. R. Sahlberg, 1823

auripes Faust, 1897
caudatus Fåhraeus, 1840
kansuanus G.A.K. Marshall, 1934
roseipes roseipes Heller, 1930
sibiricus Gyllenhal, 1834
solarii Zumpt, 1937
genus *Diglossotrox* Lacordaire, 1863
tibetanus Suvorov, 1912
genus *Megamecus* Reitter, 1903
argentatus (Gyllenhal, 1840)
urbanus (Gyllenhal, 1834)
genus *Phacephorus* Schoenherr, 1840
decipiens Faust, 1890
nebulosus (Fåhraeus, 1840)
umbratus (Faldermann, 1835)
vilis Fåhraeus, 1840
genus *Scepticus* Roelofs, 1873
noxius (Faust, 1886)
genus *Tanymecus* Germar, 1817
excursor Faust, 1890

Tribe Trachyphloeini Gistel, 1848
genus *Pseudocneorhinus* Roelofs, 1873
angustus Ren, Borovec & R. Zhang, 2019
hirsutus (Formánek, 1916)
sellatus G.A.K. Marshall, 1934

Hyperinae Lacordaire, 1863 (1848)
Tribe Hyperini Lacordaire, 1863 (1848)
genus *Brachypera* Capiomont, 1868
dauci (Olivier, 1807)
    curtithorax Pic, 1925: 6 (*Phytonomus*)
genus *Hypera* Germar, 1817
tibetana (Zaslavskij, 1966)

Lixinae Schoenherr, 1823
Tribe Cleonini Schoenherr, 1826
genus *Adosomus* Faust, 1904
melogrammus (Motschulsky, 1854)
genus *Asproparthenis* Gozis, 1886
foveocollis (Gebler, 1834)
libitinaria (Faust, 1886)
punctiventris (Germar, 1823)
salebrosicollis (Boheman, 1842)

secura (Faust, 1890)

genus *Atactogaster* Faust, 1904

inducens (Walker, 1859)

orientalis (Chevrolat, 1873)

genus *Bothynoderes* Schoenherr, 1823

declivis (Olivier, 1807)

genus *Chromonotus* Motschulsky, 1860

bipunctatus (Zoubkoff, 1829)

genus *Cleonis* Dejean, 1821

pigra (Scopoli, 1763)

genus *Conorhynchus* Motschulsky, 1860

conirostris (Gebler, 1829)

genus *Cosmogaster* Faust, 1904

cordofana (Fåhraeus, 1842)

genus *Liocleonus* Motschulsky, 1860

clathratus (Olivier, 1807)

genus *Pleurocleonus* Motschulsky, 1860

bicarinatus (Gebler, 1829)

sollicitus (Gyllenhal, 1834)

genus *Pseudocleonus* Chevrolat, 1872

dauricus (Gebler, 1829)

genus *Stephanocleonus* Motschulsky, 1860

labilis Faust, 1895

pleurocleonides (Obst, 1908)

przewalskyi Faust, 1887

suffusus Faust, 1904

suspiciosus Faust

tibetanus Suvorov, 1915

genus *Temnorhinus* Chevrolat, 1872

oryx (Reitter, 1897)

genus *Xanthochelus* Chevrolat, 1872

major (Herbst, 1784)

faunus (Olivier, 1807)

Tribe Lixini Schoenherr, 1823

genus *Lixus* Fabricius, 1801

subtilis Boheman, 1835

obliquivittis Voss, 1937

distortus Csiki, 1934

genus *Larinus* Dejean, 1821

griseopilosus Roelofs, 1873

genus *Gasteroclisus* Desbrochers des Loges, 1904

arcurostris Petri, 1912

auriculatus (C. R. Sahlberg, 1823)

binodulus (Boheman, 1835)

Molytinae Schoenherr, 1823
Tribe Cryptorhynchini Schoenherr, 1825
genus *Eucryptorrhynchus* Heller, 1937
scrobiculatus (Motschulsky, 1854)
genus *Shirahoshizo* Morimoto, 1962
squamesus Y-Q. Chen, 1991
flavonotatus (Voss, 1937)

Tribe Euderini Lacordaire, 1865
genus *Niphadonyx* Schenkling, 1932
affinis Meregalli, 2013
amplithorax Meregalli, 2013
bufo Meregalli, 2013
castaneus (Voss, 1956)
fausti Meregalli, 2013
fabbrii Meregalli, 2013
inconspicuus Meregalli, 2013
kozlovi Meregalli, 2013
laevisculptus Meregalli, 2013
limnophilus Meregalli, 2013
przewalskyi (Faust, 1887)
tara Meregalli, 2013
genus *Niphades* Pascoe, 1871
variegatus (Roelofs, 1873)
tubericollis (Faust, 1890)

Tribe Ithyporini Lacordaire, 1865
genus *Ectatorhinus* Lacordaire, 1865
adamsii Pascoe, 1872

Tribe Lepyrini W. Kirby, 1837
genus *Lepyrus* Germar, 1817
japonicus Roelofs, 1873

Tribe Mecysolobini Reitter, 1913
genus *Sternuchopsis* Heller, 1918
waltoni waltoni (Boheman, 1844)
juglans (Chao, 1980)
genus *Cylindralcides* Heller, 1918
sauteri (Heller, 1922)
genus *Merus* Gistel, 1857
dentirostris (Voss, 1932)

erro (Pascoe, 1871)
flavosignatus (Roelofs, 1875)

Tribe Molytini Schoenherr, 1823
genus *Aclees* Schoenherr, 1835
cribratus Gyllenhal, 1835
genus *Hylobius* Germar, 1817
abietis (Linnaeus, 1758)
haroldi Faust, 1882
elongatoides Voss, 1956
genus *Pimelocerus* Lacordaire, 1863
juglans (Chao, 1980)
genus *Morimotodes* Grebennikov, 2014
igori Davidian, 2019
ismene Grebennikov, 2014
magnificus Davidian, 2019

Tribe Paipalesomini G.A.K. Marshall, 1932
genus *Peribleptus* Schoenherr, 1843
foveostriatus (Voss, 1939)
scalptus Boheman, 1843

Tribe Pissodini Gistel, 1848
genus *Pissodes* Germar, 1817
punctatus Langor & R. Zhang, 1999
yunnanensis Langor & R. Zhang, 1999

Tribe Typoderini Voss, 1965
genus *Niphadomimus* Zherikhin, 1987
celaeno Grebennikov, 2014
alcyone Grebennikov, 2014
sterope Grebennikov, 2014

Platypodinae Shuckard, 1840
Tribe Platypodini Shuckard, 1840
genus *Carchesiopygus* Schedl, 1939
impariporus (Beeson, 1937)

Tribe Tesserocerini Blandford, 1896
genus *Diapus* Chapuis, 1865
quinquespinatus Chapuis, 1865
orientalis Knížek, Beaver & L.-Y. Liu, 2015

Scolytinae Latreille, 1804

Tribe Corthylini LeConte, 1876

genus *Gnatharus* Wood & Yin, 1986

tibetensis Wood & Yin, 1986

genus *Pityophthorus* Eichhoff, 1864

morosovi Spessivtsev, 1926

pini Kurentsov, 1941

genus *Pseudopityophthorus* Swaine, 1918

peregrinus Wood & Yin, 1986

Tribe Cryphalini Lindemann, 1877

genus *Cryphalus* Erichson, 1836

markangensis Tsai & C-L. Li, 1963

miyalopiceus Tsai & C-L.Li, 1963

pilosus Tsai & C-L. Li, 1963

sinoabietis Tsai & C-L. Li, 1963

saltuarius Weise, 1891

tabulaeformis Tsai & C-L. Li, 1963

lepocrinus Tsai & C-L- Li, 1963

strohmeyeri Stebbing, 1914

Tribe Crypturgini LeConte, 1876

genus *Crypturgus* Erichson, 1836

cinereus (Herbst, 1793)

pusillus (Gyllenhal, 1813)

Tribe Diamerini Hagedorn, 1909

genus *Diamerus* Erichson, 1836

fici Blandford, 1898

matangi Sampson, 1919

genus *Sphaerotrypes* Blandford, 1894

pila Blandford, 1894

tsugae Tsai & Yin, 1966

magnus Tsai & Yin, 1966

Tribe Dryocoetini Lindemann, 1877

genus *Coccotrypes* Eichhoff, 1878

cyperi (Beeson, 1929)

longior (Eggers, 1927)

nubilus (Blandford, 1894)

genus *Dryocoetes* Eichhoff, 1864

hectographus Reitter, 1913

genus *Taphrorychus* Eichhoff, 1878

picipennis (Eggers, 1926)

genus *Dryocoetiops* Schedl, 1957

nitidus (Schedl, 1942)

Tribe Ernoporini Nüsslin, 1911

genus *Eidophelus* Eichhoff, 1876

glabratus (Yin, 2001)

Tribe Hylastini LeConte, 1876

genus *Hylastes* Erichson, 1836

cunicularius Erichson, 1836

parallelus Chapuis, 1875

techangensis Tsai & Hwang, 1964

genus *Hylurgops* LeConte, 1876

major Eggers, 1944

eusulcatus Tsai & Hwang, 1964

sulcatus Eggers, 1933

longipillus Reitter, 1895

tuberculatus Eggers, 1933

Tribe Hylurgini Gistel, 1848

genus *Dendroctonus* Erichson, 1836

micans (Kugelann, 1794)

genus *Pseudoxylechinus* Wood & F.-S. Huang, 1986

tibetensis Wood & F.-S. Huang, 1986

genus *Tomicus* Latreille, 1802

minor (Hartig, 1834)

pilifer (Spessivtsev, 1919)

piniperda (Linnaeus, 1758)

Tribe Hyorrhynchini Hopkins, 1915

genus *Pseudohyorrhynchus* Murayama, 1950

blandfordi (Sampson, 1913)

Tribe Ipini Bedel, 1888

genus *Ips* DeGeer, 1775

acuminatus (Gyllenhal, 1827)

chinensis Kurentsov & Kononov, 1966

longifolia (Stebbing, 1909)

nitidus Eggers, 1933

shangrila Cognato & Sun, 2007

stebbingi Strohmeyer, 1908

sexdentatus (Boerner, 1776)

typographus (Linnaeus, 1758)

genus *Orthotomicus* Ferrari, 1867

starki Spessivtsev, 1926

suturalis (Gyllenhal, 1827)
genus *Pityogenes* Bedel, 1888
chalcographus (Linnaeus, 1761)
japonicus Nobuchi, 1974
saalasi Eggers, 1914
scitus Blandford, 1893
seirindensis Murayama, 1929
genus *Pseudips* Cognato, 2000
orientalis (Wood & Yin, 1986)

Tribe Phloeosinini Nüsslin, 1912
genus *Phloeosinus* Chapuis, 1869
aubei (Perris, 1855)
genus *Hyledius* Sampson, 1921
cribratus (Blandford, 1896)

Tribe Polygraphini Chapuis, 1869
genus *Polygraphus* Erichson, 1836
angustus Tsai & Yin, 1965
junnanicus Sokanovskiy, 1959
major Stebbing, 1903
poligraphus (Linnaeus, 1758)
rudis hexiensis Yin & F.-S. Huang, 1996
rudis rudis Eggers, 1933
szemaoensis Tsai & Yin, 1965
sinensis Eggers, 1933
verrucifrons Tsai & Yin, 1965

Tribe Scolytini Latreille, 1804
genus *Scolytus* Geoffroy, 1762
abaensis Tsai & Yin, 1962
nitidus Schedl, 1936
pilosus Yin & F.-S. Huang, 1980
pomi Yin & F.-S. Huang, 1980
querci Yin & F.-S. Huang, 1980
schevyrewi Semenov, 1902
sinopiceus Tsai, 1962

Tribe Scolytoplatypodini Blandford, 1893
genus *Scolytoplatypus* C.F.C. Schaufuss, 1891
darjeelingi Stebbing, 1914
raja Blandford, 1893
supercilliosus Tsai & Hwang, 1965

Tribe Trypophloeini Nüsslin, 1911
genus *Hypothenemus* Westwood, 1834
erectus LeConte, 1876
eruditus (Westwood, 1834)

Tribe Xyleborini LeConte, 1876
genus *Ambrosiodmus* Hopkins, 1915
asperatus (Blandford, 1895)
lewisi (Blandford, 1894)
rubricollis (Eichhoff, 1876)
genus *Anisandrus* Ferrari, 1867
apicalis (Blandford, 1894)
percristatus (Eggers, 1939)
genus *Arixyleborus* Hopkins, 1915
yakushimanus (Murayama, 1955)
genus *Cnestus* Sampson, 1911
aterrimus (Eggers, 1927)
gravidus (Blandford, 1898)
genus *Coptodryas* Hopkins, 1915
cruralis (Schedl, 1975)
genus *Cyclorhipidion* Hagedorn, 1912
improbum (Sampson, 1913)
genus *Debus* Hulcr & Cognato, 2010
emarginatus (Eichhoff, 1878)
pumilus (Eggers, 1923)
genus *Diuncus* Hulcr & Cognato, 2009
corpulentus (Eggers, 1930)
genus *Euwallacea* Hopkins, 1915
fornicatus (Eichhoff, 1868)
interjectus (Blandford, 1894)
velatus (Sampson, 1913)
genus *Hadrodemius* Wood, 1980
comans (Sampson, 1919)
pseudocomans (Eggers, 1930)
genus *Microperus* Wood, 1980
perparvus (Sampson, 1922)
genus *Webbia* Hopkins, 1915
pabo Sampson, 1922
genus *Xyleborinus* Reitter, 1913
saxesenii (Ratzeburg, 1837)
genus *Xylosandrus* Reitter, 1913
brevis (Eichhoff, 1877)
crassiusculus (Motschulsky, 1866)
germanus (Blandford, 1894)

mancus (Blandford, 1898)

Tribe Xyloterini LeConte, 1876

genus *Indocryphalus* Eggers, 1939

intermedius (Sampson, 1913)

genus *Trypodendron* Stephens, 1830

lineatum (Olivier, 1800)

proximum (Niisima, 1909)

signatum (Fabricius, 1792)
